# Mutual interference between memory encoding and motor skills: the influence of motor expertise

**DOI:** 10.3389/fpsyg.2023.1196978

**Published:** 2023-12-15

**Authors:** Annalena Monz, Kathrin Morbe, Markus Klein, Sabine Schaefer

**Affiliations:** Institute of Sport Sciences, Saarland University, Saarbrücken, Germany

**Keywords:** memory encoding, dual-tasking, motor performance, expertise, martial arts, rowing

## Abstract

In cognitive–motor dual-task situations, the extent of performance decrements is influenced by the attentional requirements of each task. Well-learned motor skills should be automatized, leading to less interference. This study presents two studies combining an episodic memory encoding task with well-practiced motor tasks in athletes. Study 1 asked 40 rowers (early teenagers to middle adulthood) to row on ergometers at slow or fast speeds. In study 2, Taekwondo athletes (*n* = 37) of different skill levels performed a well-practiced sequence of martial arts movements. Performing the motor task during encoding led to pronounced performance reductions in memory in both studies, with costs of up to 80%. Cognitive costs were even larger when rowing with the fast compared to the slow speed in study 1. Both studies also revealed decrements in motor performances under dual-task conditions: Rowing became slower and more irregular (study 1), and the quality of the Taekwondo performance was reduced. Although higher-level athletes outperformed others in motor skills under single-task conditions, proportional dual-task costs were similar across skill levels for most domains. This indicates that even well-practiced motor tasks require cognitive resources.

## Introduction

The interplay of cognitive and motor performance is relevant in many everyday situations. For example, people are engaged in a conversation while walking, or they try to remember their grocery shopping list while riding their bicycle. Performing a cognitive and a motor task concurrently often leads to performance decrements (for reviews, see Schaefer, 2014; Koch et al., 2018; Broeker et al., 2022). Classic accounts on the nature of such deficits either propose a limited central resource that has to be shared between the two tasks (Kahneman, 1973), a limited pool of processing resources (Wickens, 2008), or processing stages that can only be operated sequentially by each task (Pashler, 1994).

While most cognitive–motor dual-task studies have been conducted with everyday motor activities such as walking, more challenging motor tasks from a sports context may also be used. In this context, experience with motor skills should enable athletes to reduce their dual-task costs. Theories on motor skill learning predict that automatized tasks require less attention (Fitts and Posner, 1967; Adams, 1971; Gentile, 1972). Dual-process theories propose that human behavior requires two different types of processes. Type 1 processes are independent of attentional control and support the execution of well-learned tasks. Type 2 processes depend on cognitive processing resources, such as attention and working memory capacity (Shiffrin and Schneider, 1977; Kahneman, 2011; Evans and Stanovich, 2013; Furley et al., 2015; Furley and Wood, 2016). They “take over” when the situation is complex and requires higher-order cognitive processes. If intensive experience with a motor skill leads to its automatization, skilled athletes should show smaller dual-task costs than novices when performing a cognitive task concurrently with their motor skills.

For sport-specific task combinations, studies in golf putting, baseball, rugby, soccer, track and field, ice hockey, climbing, or gymnastics reported the predicted performance advantages of experts (Leavitt, 1979; Parker, 1981; Abernethy, 1988; Castiello and Umiltà, 1988; Smith and Chamberlin, 1992; Vuillerme et al., 2001; Beilock et al., 2002a,b, 2004; Gray, 2004; Vuillerme and Nougier, 2004; Gabbett et al., 2011; Green and Helton, 2011; Gabbett and Abernethy, 2013; Darling and Helton, 2014). However, there is considerable variation in study designs concerning the use of discrete actions (e.g., reaction-time tasks and throwing a ball at a target) vs. continuous tasks (e.g., running, maintaining posture, skating, and working memory updating). In addition, many studies instructed participants to maintain their motor task performance under dual-task conditions and used the cognitive task primarily to disturb the execution of the motor task (in the sense of a “secondary task”). Single-task baseline performance in cognition has often not been measured at all. This makes it difficult to get a full picture of dual-task deficits. We argue that performance changes from single- to dual-task conditions should be measured for both task domains (Li et al., 2005; Schaefer, 2014; Plummer and Eskes, 2015). Proportional dual-task costs express the performance decrements in relation to each individual’s baseline performance (Somberg and Salthouse, 1982). This allows for a comparison of performance decrements across task domains and groups and can reveal reciprocal dual-task effects (Plummer and Eskes, 2015) and task prioritization strategies (Li et al., 2005).

Taking these considerations into account, a study by Schaefer and Scornaienchi (2019) asked young expert and novice table tennis players to perform a working memory task while returning balls from a ball machine. The cognitive task, 3-back, was a continuous working memory updating task. Participants were presented with a stream of numbers and had to compare the current number to the number presented three positions earlier in the sequence. Stimulus presentations of balls and numbers were varied within subjects by either presenting a ball and a number in the same time window or one after the other, avoiding central or peripheral processing bottlenecks (Abernethy, 1988; Pashler, 1994). There were no differences between experts and novices in their 3-back performances under single-task conditions. However, novices showed higher cognitive dual-task costs. For table tennis (number of balls returned successfully), experts outperformed novices already in the single task. Across both task domains, experts consistently showed costs of about 10%, while novices showed costs between 30% and 50%. However, concurrent vs. alternating stimulus presentation did not influence dual-task costs in this study.

Schaefer and Amico (2022) expanded these findings in another sample of table tennis experts and novices. In addition to 3-back and table tennis returns (timed tasks), each subject also performed the task of counting backward in steps of 7 and table tennis serves (self-initiated tasks). All combinations of cognitive and motor tasks were assessed in a within-subjects design under single- and dual-task conditions. It was assumed that self-initiated tasks should increase dual-task costs since the scheduling of the responses requires attentional resources. As in the previous study, dual-task costs of novices were considerably higher (35%) than those of experts, who did not show costs (−1%). Costs for self-initiated tasks were higher only in the experts, while novices showed a tendency to reduce their dual-task costs for self-initiated tasks. The authors attribute this to the psychometric properties of the underlying tasks since timed tasks were specified by a fixed number of targets and responses.

Another recent set of studies by Amico and Schaefer (2022) used tennis instead of table tennis as the motor task and focused on expert vs. intermediate players instead of novices. For the tennis task, participants had to return balls to a target field. Two different cognitive tasks were used: a 3-back working memory task and a vocabulary-learning task (episodic memory). Dual-tasking led to performance reductions in both cognitive tasks, but the accuracy of tennis returns remained stable under cognitive challenge. Skilled tennis players showed a task-prioritization strategy in favor of the tennis task in the dual-task situation (see also Plummer and Eskes, 2015). Intermediate players showed higher overall dual-task costs than experts in the study with 3-back. However, the group differences in dual-task costs did not reach significance when subjects were asked to learn vocabulary, possibly due to less pronounced expertise differences between the groups.

A recent review by Tomporowski and Qazi (2020) on cognitive–motor interference effects on declarative memory suggests that the type of concurrent motor task and the characteristics of the performer may influence dual-task performance patterns. The authors summarize different theoretical assumptions on the dual-task interplay between physical exercise and cognition. According to arousal theories, the intensity of the exercise will influence whether it exerts facilitative or inhibitory effects on concurrent memory encoding. With low or intermediate intensities, an acute bout of physical exercise can be beneficial for memory performance (Schmidt-Kassow et al., 2010, 2013, 2014; McMorris and Hale, 2012; Roig et al., 2013, 2016; Loprinzi et al., 2019). This may be due to changes in physiological arousal and the release of neurotransmitters and nerve growth factors (see reviews by McMorris, 2016; Loprinzi et al., 2019). However, if the concurrent exercise is too intense, cognitive performance is likely to suffer (see also Dietrich, 2006; Dietrich and Audiffren, 2011).

On the other hand, attention theories would predict that any type of motor task that requires attention should lead to decrements in the concurrent cognitive activity, namely the encoding of the to-be-remembered words. Note that theories on motor skill learning and dual-process theories (Adams, 1971; Gentile, 1972; Shiffrin and Schneider, 1977; Furley et al., 2015) would make similar predictions, with the additional assumption that well-learned tasks require fewer attentional resources.

The current article presents two studies on the influence of motor skill level in cognitive–motor dual-tasking. Study 1 asked rowers in four different ability groups (teenagers to middle-aged adults) to row on ergometers with two different speeds, easy and hard. For rowing, a single generalized motor program is established and performed using online interoceptive and exteroceptive feedback. Rowing speeds were calibrated to each individual’s performance level. Study 2 recruited Taekwondo athletes of three different expertise levels. Taekwondo, as a sport, makes use of an elaborate grading system, as reflected by the color of the athlete’s belt (black being the highest). The motor task consisted of performing a pre-specified elaborate sequence of martial art movements (“practicing forms”), similar to a dance, with movement quality being rated by expert judges. Forms are sets of prearranged movements to simulate interactions with imaginary opponents (Minarik, 2014, p. 33; Johnson, 2019, p. 1,650).

The cognitive task of both studies is an episodic memory task, the Method-of-Loci. For this task, participants are instructed to use a pre-specified sequence of location cues to encode word lists. The task has been used successfully in several cognitive–motor dual-task studies in different age groups (Kliegl et al., 1990; Li et al., 2001; Schaefer et al., 2008; Amico and Schaefer, 2020, 2022). In the current set of studies, participants perform the cognitive task under single-task conditions while simultaneously rowing on the ergometer (study 1) or performing the martial arts movements (study 2).

The motor and cognitive tasks of the current studies are continuous. While rowing is a cyclic motor skill requiring primarily strength and endurance, performing Taekwondo forms demands timing, movement accuracy, and coordination. Participants are asked to work on the tasks for a prolonged period. Performances and costs are presented on a macro-level, aggregating over several responses (see also Koch et al., 2018, p. 561). The paradigm is, therefore, not suited to answer specific questions about the scheduling of processing steps of each task or about central or peripheral bottlenecks (Pashler, 1994; Hommel, 2020; Huestegge and Strobach, 2021). The studies had not been planned to investigate gender differences since previous dual-task studies often did not find interactions between dual-task costs and gender (Hirsch et al., 2019).

To summarize, study 1 (rowing) investigates whether rowing in two different intensities diminishes memory performance and whether motor expertise moderates this influence. Study 2 assesses whether practicing a form in Taekwondo during memory encoding leads to performance decrements. For both studies, we predict that higher-level athletes are more successful in keeping up their cognitive performances under dual-task conditions. Since single- and dual-task performances are assessed repeatedly for each task involved, we can also investigate whether rowing speed and rowing regularity suffer from dual-tasking (study 1) and whether practicing Taekwondo forms is performed less well under dual-task conditions (study 2). In addition, the calculation of proportional dual-task costs allows for a comparison of findings across groups, tasks, and studies.

## Study 1: Rowing

### Methods

#### Participants

Participants were recruited from a local rowing club in Saarbrücken. The club’s training groups differed in performance level, with the younger teenagers (teens 1; 5 men, 5 women) being relative beginners, the older teenagers having more experience in rowing (teens 2; 10 men), the young adults belonging to an elite performance group that regularly takes part in competitions at the regional and national levels (young adults; 8 men, 2 women), and the middle-aged adults (4 men, 6 women) being master athletes who do not compete on a regular basis anymore. Table 1 presents the background information for each group. All participants had normal or corrected-to-normal vision and hearing and gave informed consent to the study. The study was approved by the ethics committee of Saarland University.

**Table 1:** Background information about the four groups of Study 1 (rowing).

Age group		Teens 1	Teens 2	Young adults	Middle-aged adults	*p*-values for follow-up comparisons
*N* (men/women)		10 (5/5)	10 (10/0)	10 (8/2)	10 (4/6)	
Age (years)	*M*	12.8	16	20.5	55.5	
	*SD*	2.5	0.3	0.7	1.7	
	*Range*	12–14	14–18	17–24	48–63	
Entertainment (none/music/radio)		7/2/1	3/5/2	1/9/0	6/2/2	
Rowing experience (years)	*M*	1.8	3.4	6.8	12.0	Teens 1 vs. Teens 2 *p* = 1.000
						Teens 1 vs. Young *p* = 0.058
	*SD*	0.6	1.1	3.0	4.7	Teens 1 vs. MA *p* < 0.001
						Teens 2 vs. Young *p* = 0.412
						Teens 2 vs. MA *p* < 0.001
						Young vs. MA *p* = 0.041
Weekly rowing (minutes)	*M*	318	395	616	353	Teens 1 vs. Teens 2 *p* = 1.000
						Teens 1 vs. Young *p* = 0.007
	*SD*	126	190	264	151	Teens 1 vs. MA *p* = 1.000
						Teens 2 vs. Young *p* = 0.081
						Teens 2 vs. MA *p* = 1.000
						Young vs. MA *p* = 0.023
Prescribed target time “Easy”	*M*	195	138	131	175	Teens 1 vs. Teens 2 *p* < 0.001
						Teens 1 vs. Young *p* < 0.001
	*SD*	27	16	10	20	Teens 1 vs. MA *p* = 0.154
						Teens 2 vs. Young *p* = 1.000
						Teens 2 vs. MA *p* < 0.001
						Young vs. MA *p* < 0.001
Prescribed target time “Hard”	*M*	167	120	107	145	Teens 1 vs. Teens 2 *p* < 0.001
						Teens 1 vs. Young *p* < 0.001
	*SD*	29	13	7	22	Teens 1 vs. MA *p* = 0.107
						Teens 2 vs. Young *p* = 1.000
						Teens 2 vs. MA *p* = 0.033
						Young vs. MA *p* < 0.001
Digit symbol test (correct items)	*M*	55.20	59.10	62.40	48.50	Teens 1 vs. Teens 2 *p* = 1.000
						Teens 1 vs. Young *p* = 0.639
						Teens 1 vs. MA *p* = 0.793
	*SD*	8.13	8.66	12.55	8.92	Teens 2 vs. Young *p* = 1.000
						Teens 2 vs. MA *p* = 0.119
						Young vs. MA *p* = 0.017

Target times are expressed in “seconds to row 500 m.” The prescribed times were calculated based on each athlete’s usual performance. “Entertainment” refers to the activities that participants usually do during their rowing training sessions (none = no concurrent activity, listening to music, listening to the radio). Follow-up analyses for significant group main effects are Bonferroni-corrected. “MA” refers to “Middle-Aged Adults.”

A statistical power analysis was performed for sample size estimation (GPower 3.1; Faul et al., 2007). Within-subjects designs and highly reliable measures increase statistical power (Brysbaert and Stevens, 2018; Rouder and Haaf, 2018; Zwaan et al., 2018; Brysbaert, 2019). We expected age- and expertise-related influences on dual-task decrements to be large (Leavitt, 1979; Schaefer and Scornaienchi, 2019; Amico and Schaefer, 2022; Schaefer and Amico, 2022). We conducted a power calculation using the G*3 Power software (Faul et al., 2007), with a significance level of 0.05. The power analysis focused on the interaction effect of expertise/age group and single- vs. dual-task performance decrements. We assumed the correlation among repeated measures to be high (*r* = 0.65; see also Schaefer and Scornaienchi, 2019). The analysis indicated that a medium-to-large effect size of *f* = 0.3 for four groups and two measurement occasions (single- vs. dual-tasking) would lead to an actual power of 0.96 with a total sample size of 40 participants.

#### Procedure

Each participant took part in four group sessions with up to five participants. Before the first session, participants had familiarized themselves with the MoL memory strategy by watching an educational video recorded by the senior author. The video explains how the method works and presents an example trial of six location-word combinations. In session 1, after assessing the demographic information, participants performed one single-task MoL trial while sitting. They also performed a rowing trial in the easy condition for 180 s, without any concurrent task. Session 2 (easy speed) and session 3 (hard speed) assessed the dual-task performances by presenting the MoL task in the second half of the rowing trial. In session 4, one additional MoL single-task trial was administered, and participants also performed an additional rowing trial in the easy condition without any concurrent task.

#### Apparatus and experimental tasks

##### Background measure

Perceptual-motor speed was measured with the Digit-Symbol Substitution task (Wechsler, 1981).

##### Cognitive task: method of loci

The method of loci task (MoL) is a well-established memory strategy to encode word lists (Kliegl et al., 1990; Li et al., 2001; Schaefer et al., 2008; Amico and Schaefer, 2020). To-be-encoded words are concrete objects, and they are encoded by generating a mental image of the object at a specific location. A predefined sequence of 20 location cues was used in the current study. The locations are part of every apartment (e.g., bed, window, table, and chair). The to-be-learned words were taken from Brehmer et al. (2004). They consisted of concrete German nouns that can be easily imagined, such as objects, animals, or professions. In each trial, participants heard lists of 20 location-word combinations presented auditorily with an inter-stimulus interval of 5 s. The instruction was to encode the to-be-learned word by combining it with the respective location cue via mental imagery. Participants were encouraged to include object size, sound, touch, emotions, or movement, depending on their personal preferences. For example, when encoding the word “spider” at the location “table,” a participant could imagine a huge hairy spider crawling over the table. The current study used “cued” encoding and recall conditions, presenting the location cue with the word during encoding and the list of locations in the correct order for recall. Immediately after the last word was presented, participants wrote down the remembered words at the corresponding location cue on their answer sheets. There was no time limit for recall. The dependent variable for MoL was the sum of correctly remembered words at the correct location.

The encoding for the MoL task was performed while sitting (single-task condition) or rowing (dual-task condition).

##### Rowing task

Rowing took place on a rowing ergometer (PM5; Concept Two; Morrisville, Vermont, United States). All participants were accustomed to these ergometers because they are regularly used for training purposes in their rowing club, with the instruction to keep up a specific rowing speed for longer time periods.

The display of the ergometer presents the following information: total rowing time, current time to row 500 m, average time to row 500 m for the entire training session, and current rate of strokes. The largest item on the display is the *current time to row 500 m*, and participants are familiar with using this value to calibrate their rowing intensity. Based on the usual performance level of each participant during the previous winter training period and the feedback from the coach, the experimenter individually calculated two rowing intensities for each person: an easy speed and a hard speed. Heart rates for each condition are presented in Supplementary material 1. Table 1 presents the target times for easy and hard rowing. Each rowing trial lasted 180 s, and the main dependent variables for rowing quality were the average time taken to row 500 m and the *SD* of the time taken to row 500 m (rowing regularity). Since rowing performance was recorded for each 10-s segment of the trial, we could also investigate how strongly participants fluctuated in their rowing performance over time by plotting the rowing performances by segment (see Supplementary material 2).

##### Single- and dual-task setting

In the single-task trials of MoL, participants performed the cognitive task while sitting on the rowing ergometer without any concurrent motor activity. Rowing trials lasted 180 s. Some rowing trials consisted of two parts. In the first half of the trial, no concurrent cognitive stimuli were presented (single-task rowing). In the second half, participants were presented with the location-word combinations (dual-task rowing). They were instructed to keep up their rowing speed while concurrently encoding the location-word pairs. Immediately following the last pair, the rowing trial ended. Participants listed all the words they could remember, at the correct location, on their answer sheets. There was no time limit for recall.

#### Overview of analysis

The statistical analysis was conducted via IBM SPSS Statistics 25 (IBM Corporation, Armonk, NY, United States). The reliability for MoL and rowing was tested by calculating Cronbach’s alpha. Mixed-design ANOVAs with group (4) as a between-subjects factor and condition (single- vs. dual-tasking) as a within-subjects factor were conducted. For rowing, rowing speed (2: easy vs. hard) was included as an additional within-subjects factor. Furthermore, dual-task costs (DTCs) were calculated, expressing performance reductions under dual-task conditions as a percentage of each individual’s single-task performance, with the following formula:


$$DTC\;\mathrm{in}\%=\frac{\left|\mathrm{mean}\;\mathrm{score}\;\left(\mathrm{single}\right)-\mathrm{mean}\;\mathrm{score}\;\left(\mathrm{dual}\right)\right|}{\mathrm{mean}\;\mathrm{score}\;\left(\mathrm{single}\right)}\cdot 100$$

DTCs for each task domain were analyzed with univariate or mixed-design ANOVAs. For all ANOVAs, *F* values and generalized Eta square values ( $\eta_{G}^{2})$ or partial Eta square values ( $\eta_{p}^{2})$ for effect sizes are reported. To interpret statistical significance, the alpha level *α* = 0.05 was used. Significant main effects and interactions were further investigated by follow-up analyses with Bonferroni correction.

All data and analysis code can be made available upon request to the senior author. Data were analyzed using IBM SPSS Statistics 25 (IBM Corporation, Armonk, NY, United States). The study’s design and analyses were not pre-registered.

### Results

#### Participant background information

Table 1 shows that the samples differed concerning their average age, the years that they had been rowing [*F*(3, 36) = 12.31, *p* < 0.001, *η^2^p* = 0.506], the minutes that they spent rowing each week [*F*(3, 36) = 4.97, *p* = 0.005, *η^2^p* = 0.239], and their Digit Symbol Substitution scores [*F*(3, 36) = 3.77, *p* = 0.019, *η^2^p* = 0.239]. The Digit Symbol scores corresponded to samples of other representative studies (see Schmiedek et al., 2010), with young adults outperforming middle-aged adults.

#### Method of loci task

**Figure 1:**
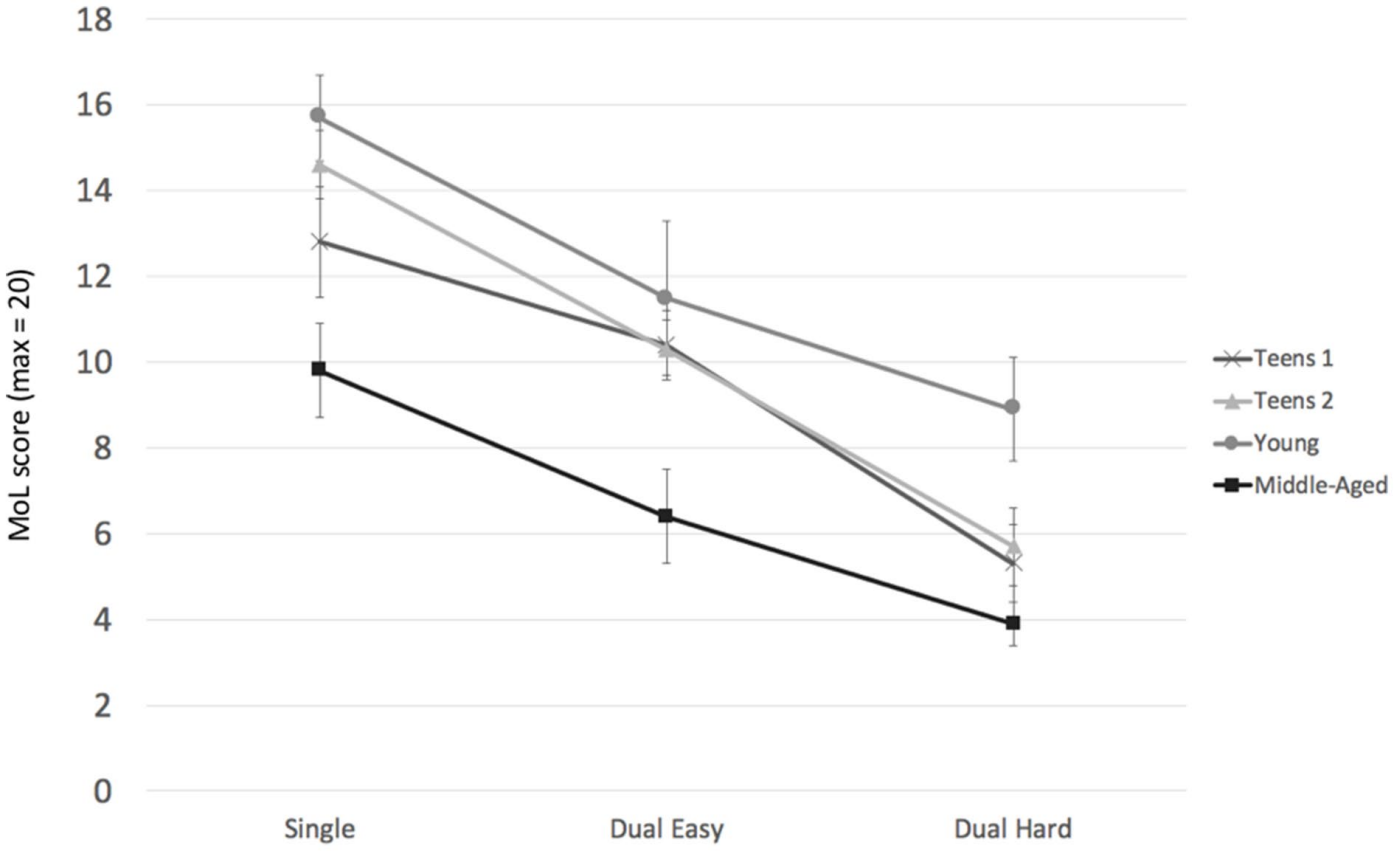
Memory performances by single- and dual-task condition and age group, study 1 (Rowing). Participants encoded the word-location pairs while sitting in the “Single” Condition. In the “Dual Easy” condition, participants were rowing at an easy speed while encoding the location-word pairs. The rowing speed was faster in the “Dual Hard” condition. Error bars = SE mean.

The reliability coefficient based on the four MoL trials was excellent (*α* = 0.930), indicating that the interindividual differences in memory performance remained stable over consecutive trials. Results are presented in Figure 1. Note that performances of the two single-task MoL trials from session 1 and session 4 were averaged to control for practice effects over the course of the study. The mixed-design ANOVA with group (4: teens 1, teens 2, young, and middle-aged) as a between-subjects factor and condition (3: single-task, dual-easy, dual-hard) as a within-subjects factor showed a significant main effect of single- vs. dual-tasking, *F*(2, 72) = 171.00; *p* < 0.001; $\eta_{G}^{2}$ = 0.466. MoL performances decreased linearly from single-task to dual-task with easy and hard rowing speeds. The main effect of group also reached significance, *F*(3, 36) = 3.34, *p* = 0.004, $\eta_{G}^{2}$ = 0.267, and there was a significant interaction of group and condition, *F*(6, 72) = 2.25, *p* = 0.048, $\eta_{G}^{2}$ = 0.033.

To follow-up the significant interaction of group and condition, ANOVAs for each of the three conditions revealed a significant main effect of group for MoL single-task performances, *F*(3, 36) = 6.13, *p* = 0.002, $\eta_{p}^{2}$ = 0.338. Bonferroni-corrected comparisons revealed significant differences between older teenagers and middle-aged adults (*p* = 0.015, $M_{\mathrm{Diff}}$ = 4.800, 95%-CI [0.69, 8.91]), as well as between young and middle-aged adults (*p* = 0.002, $M_{\mathrm{Diff}}$ = 5.900, 95%-CI [1.79, 10.01]). MoL performances also differed between groups when concurrently rowing with the easy speed, *F*(3, 36) = 3.50, *p* = 0.025, $\eta_{p}^{2}$ = 0.226. The only significant difference in the Bonferroni-corrected multiple comparisons was between young and middle-aged adults (*p* = 0.028, $M_{\mathrm{Diff}}$ = 5.10, 95%-CI [0.38, 9.82]). While rowing with the hard speed, MoL performances again differed significantly across groups, *F*(3, 36) = 5.18, *p* = 0.004, $\eta_{p}^{2}$ = 0.302, due to significant differences between young and middle-aged adults (*p* = 0.003, $M_{\mathrm{Diff}}$ = 5.00, 95%-CI [1.34, 8.66]). Across all analyses, young adults showed the highest MoL scores, and middle-aged adults showed the lowest scores.

#### Rowing performance: mean time to row 500 m

The reliability coefficient based on the rowing performances in the four segments of the rowing trials (single- and dual-task segments of the easy and hard trials) was excellent (*α* = 0.997).

**Figure 2:**
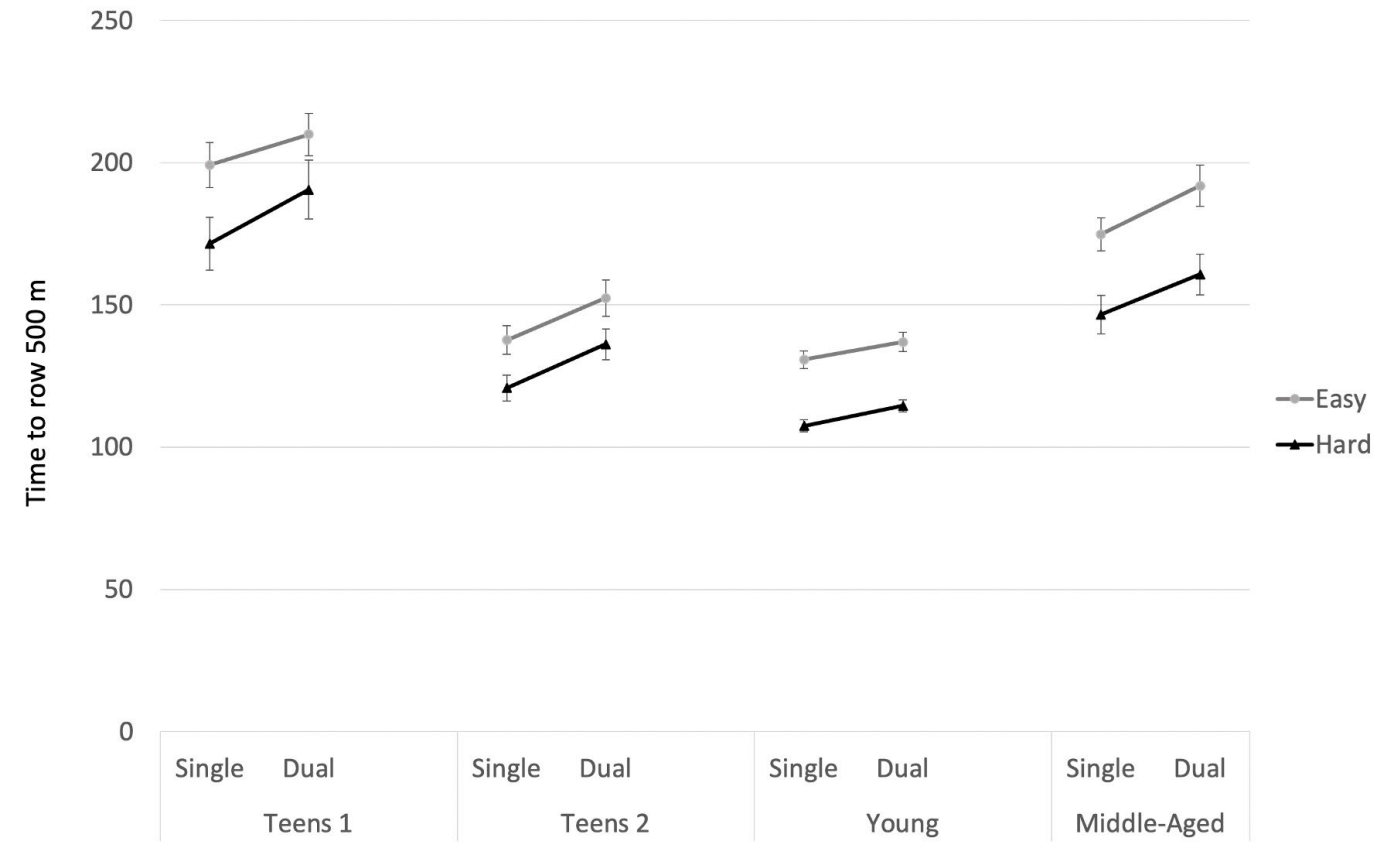
Rowing Times by task difficulty, single- vs. dual-task condition, and age group, study 1. Rowing for the “Single” conditions was assessed in the first part of the respective trial when no location-word pairs were presented. The “Dual” condition was assessed in the second half of the trial while encoding the word-location pairs. Error bars = SE mean.

To compare the difference in performance from single- to dual-tasking, a mixed-design ANOVA was calculated, with rowing intensity (2: easy vs. hard) and single- vs. dual-tasking (2: first vs. second part of the respective trial) as within-subjects factors and group (4: teens 1, teens 2, young, and middle-aged) as a between-subjects factor. The main effect of rowing intensity reached significance, *F*(1, 36) = 177.92; *p* < 0.001; $\eta_{G}^{2}$ = 0.272. Participants rowed faster in the hard rowing intensity. There was no interaction of rowing intensity and group, *F*(3, 36) = 2.40; *p* = 0.084; $\eta_{G}^{2}$ = 0.015. There was also a significant main effect of dual-tasking, *F*(1, 36) = 217.93; *p* < 0.001; $\eta_{G}^{2}$ = 0.106, due to performance reductions under dual-task conditions. Dual-tasking interacted with group, *F*(3, 36) = 5.87; *p* = 0.002; $\eta_{G}^{2}$ = 0.010. While the two-way interaction of rowing intensity and single- vs. dual-tasking failed to reach significance, *F*(1, 36) = 1.85; *p* = 0.183; $\eta_{G}^{2}$ = 0.001, there was a significant three-way interaction of rowing intensity, single- vs. dual-tasking, and group, *F*(3, 36) = 3.58; *p* = 0.023; $\eta_{G}^{2}$ = 0.003. The main effect of group also reached significance, *F*(3, 36) = 28.03; *p* < 0.001; $\eta_{G}^{2}$ = 0.677. Young adults showed the fastest rowing speeds, and the younger teenagers showed the slowest speeds. Figure 2 presents the pattern of findings.

For follow-up analyses, we conducted ANOVAs with rowing intensity (2) and single- vs. dual-tasking (2) for each age group separately. Table 2 presents the results. The main effects of rowing intensity reached significance in each of the four groups, with faster rowing speeds in the “hard” compared to the “easy” condition. In addition, rowing while encoding the MoL words led to a deterioration of rowing performance in each group. However, the interaction of rowing intensity and single- vs. dual-tasking did not reach significance in any of the groups due to the Bonferroni correction of the *p*-values to *p* = 0.0125.

**Table 2:** Results of follow-up analyses for the mixed-design ANOVA on mean times to row 500 m.

Age group	Teenager 1	Teenager 2	Young adults	Middle-aged adults
Main effect rowing intensity	*F*(1, 9) = 19.43	*F*(1, 9) = 29.59	*F*(1, 9) = 112.70	*F*(1, 9) = 148.57
	*p* = 0.002	*p* < 0.001	*p* < 0.001	*p* < 0.001
	$\eta_{p}^{2}$ = 0.683	$\eta_{p}^{2}$ = 0.767	$\eta_{p}^{2}$ = 0.926	$\eta_{p}^{2}$ = 0.943
Main effect single vs. dual	*F*(1, 9) = 49.19	*F*(1, 9) = 49.20	*F*(1, 9) = 69.78	*F*(1, 9) = 88.18
	*p* < 0.001	*p* < 0.001	*p* < 0.001	*p* < 0.001
	$\eta_{p}^{2}$ = 0.845	$\eta_{p}^{2}$ = 0.845	$\eta_{p}^{2}$ = 0.886	$\eta_{p}^{2}$ = 0.907
Interaction intensity × single–dual	*F*(1, 9) = 7.58	*F*(1, 9) = 0.08	*F*(1, 9) = 0.13	*F*(1, 9) = 1.47
	*p* = 0.022	*p* = 0.784	*p* = 0.724	*p* = 0.257
	$\eta_{p}^{2}$ = 0.457	$\eta_{p}^{2}$ = 0.009	$\eta_{p}^{2}$ = 0.014	$\eta_{p}^{2}$ = 0.140

#### Rowing performance: SDs of time to row 500 m

**Figure 3:**
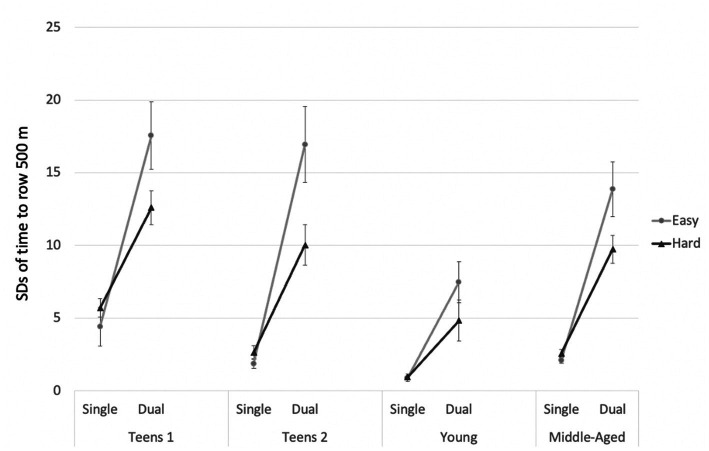
SDs of rowing times for Study 1. Error bars = SE mean.

To address the fluctuations in rowing speed, we also investigated the standard deviations (*SD*s) of rowing times (per 500 m) for rowing under single- and dual-task conditions. Figure 3 presents the pattern of findings.

A mixed-design ANOVA was calculated, with rowing intensity (2: easy vs. hard) and single- vs. dual-tasking (2: first vs. second segment of the respective trial) as within-subjects factors and group (4: teens 1, teens 2, young, and middle-aged) as a between-subjects factor. The main effect of rowing intensity reached significance, *F*(1, 36) = 17.16; *p* = 0.002; $\eta_{G}^{2}$ = 0.236. Rowing was less regular in the easy speed. There was no interaction of rowing intensity and group, *F*(3, 36) = 0.40; *p* = 0.756; $\eta_{G}^{2}$ = 0.007. The was also a significant main effect of dual-tasking, *F*(1, 36) = 199.38; *p* < 0.001; $\eta_{G}^{2}$ = 0.574, due to increased rowing irregularity under dual-task conditions. Dual-tasking interacted with group, *F*(3, 36) = 4.26; *p* = 0.012; $\eta_{G}^{2}$ = 0.323. The two-way interaction of rowing intensity and single- vs. dual-tasking also reached significance, *F*(1, 36) = 15.90; *p* < 0.001; $\eta_{G}^{2}$ = 0.105, but there was no significant three-way interaction of rowing intensity, single- vs. dual-tasking, and group, *F*(3, 36) = 0.64; *p* = 0.592; $\eta_{G}^{2}$ = 0.014. The main effect of group also reached significance, *F*(3, 36) = 16.09; *p* < 0.001; $\eta_{G}^{2}$ = 0.269. Young adults showed the most regular rowing of all groups.

For follow-up analyses, we conducted ANOVAs with rowing intensity (2) and single- vs. dual-tasking (2) for each age group separately. Table 3 presents the results. Due to the Bonferroni correction of the *p*-values to *p* = 0.0125, the main effects of rowing intensity did not reach significance in any of the four groups. However, rowing while encoding the MoL words led to a significantly less consistent rowing pattern, with more fluctuations in each group. The interaction of rowing intensity and single- vs. dual-tasking only reached significance in older teenagers.

**Table 3:** Results of follow-up analyses for the mixed-design ANOVA on SDs to row 500 m.

Age group	Teenager 1	Teenager 2	Young adults	Middle-aged adults
Main effect rowing intensity	*F*(1, 9) = 1.67	*F*(1, 9) = 7.81	*F*(1, 9) = 1.04	*F*(1, 9) = 3.43
	*p* = 0.229	*p* = 0.021	*p* = 0.334	*p* = 0.097
	$\eta_{p}^{2}$ = 0.156	$\eta_{p}^{2}$ = 0.465	$\eta_{p}^{2}$ = 0.104	$\eta_{p}^{2}$ = 0.276
Main effect single vs. dual	*F*(1, 9) = 53.42	*F*(1, 9) = 44.47	*F*(1, 9) = 65.30	*F*(1, 9) = 66.66
	*p* < 0.001	*p* < 0.001	*p* < 0.001	*p* < 0.001
	$\eta_{p}^{2}$ = 0.856	$\eta_{p}^{2}$ = 0.832	$\eta_{p}^{2}$ = 0.879	$\eta_{p}^{2}$ = 0.881
Interaction intensity × single–dual	*F*(1, 9) = 3.13	*F*(1, 9) = 9.98	*F*(1, 9) = 1.19	*F*(1, 9) = 5.49
	*p* = 0.111	*p* = 0.012	*p* = 0.304	*p* = 0.044
	$\eta_{p}^{2}$ = 0.258	$\eta_{p}^{2}$ = 0.526	$\eta_{p}^{2}$ = 0.117	$\eta_{p}^{2}$ = 0.379

Results for each 10-s segment of a trial are presented in Supplementary material 2. An analysis of the rowing-only trials in the easy condition (assessed in sessions 1 and 4) shows that performance reductions in dual-task rowing were not due to fatigue effects but due to cognitive load (see Supplementary material 2 for details).

#### Dual-task costs

To compare the differences in performance between the four groups across the single- and dual-task conditions for the motor domain (rowing easy and rowing hard) and the cognitive domain (MoL), percentage scores for the dual-task costs (DTCs) have been calculated.

**Figure 4:**
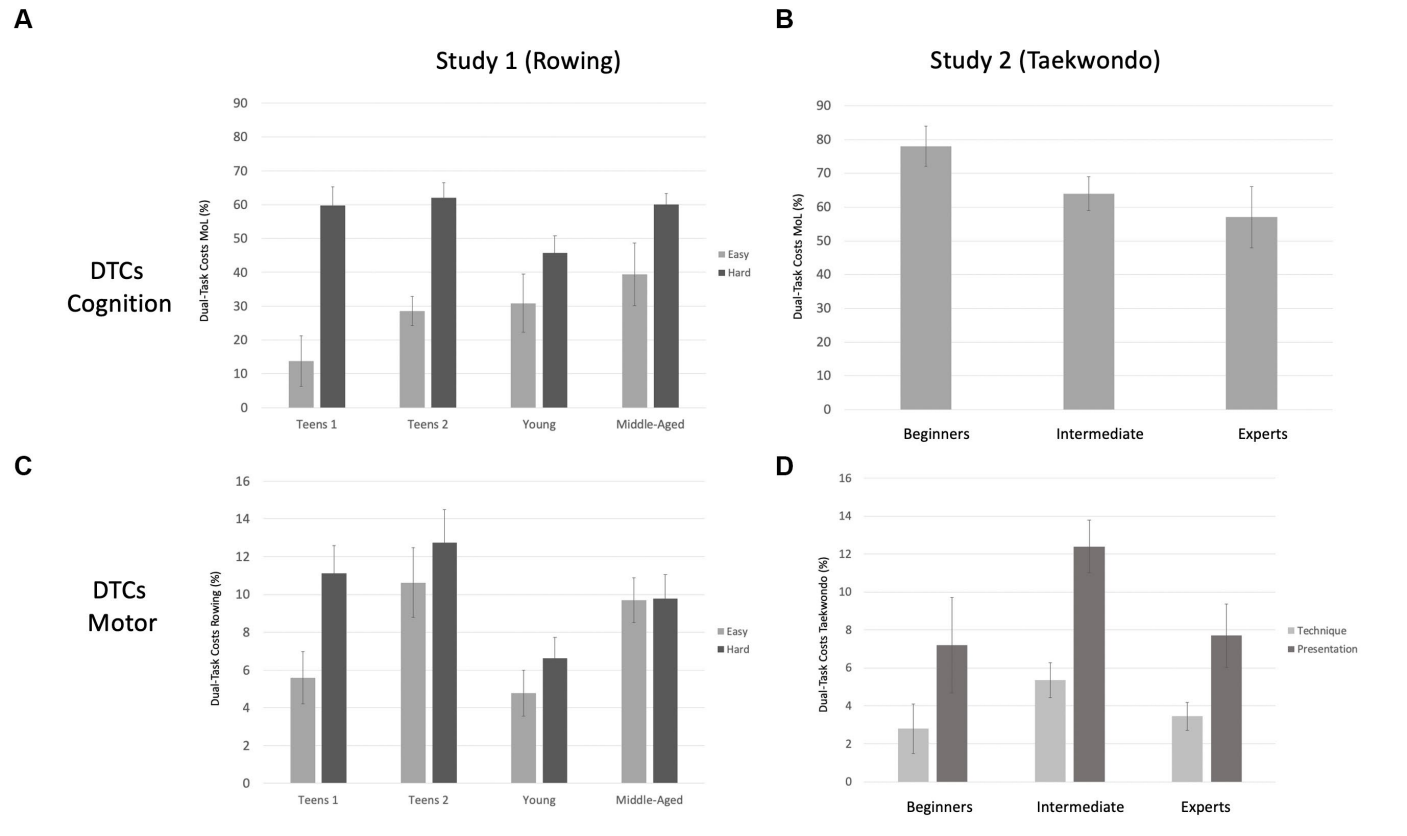
Dual-task costs for both studies. The left-hand side of the figure presents the DTCs for study 1 **(A,C)**, and the right-hand side presents the DTCs for study 2 **(B,D)**. Costs in cognition (MoL) are presented in the first row, and costs for motor tasks (rowing in study 1, Taekwondo in study 2) are presented in the second row. Error bars = SE mean.

The left-hand side of Figure 4 illustrates the DTCs for this study. DTCs for MoL are presented in Figure 4A, and DTCs for rowing are presented in Figure 4C.

For the cognitive DTCs, a mixed-design ANOVA with rowing intensity (2: easy vs. hard) as a within-subjects factor and group (4: teens 1, teens 2, young, and middle-aged) as a between-subjects factor was calculated. The main effect of rowing intensity reached significance, *F*(1, 36) = 64.37; *p* < 0.001; $\eta_{G}^{2}$ = 0.364. Cognitive costs were higher when rowing at a fast speed. There was also a significant interaction of rowing intensity and group, *F*(3, 36) = 3.74; *p* = 0.020; $\eta_{G}^{2}$ = 0.091. The main effect of group did not reach significance, *F*(3, 36) = 1.35; *p* = 0.275; $\eta_{G}^{2}$ = 0.071.

Paired sample t-tests were conducted within each age group to follow up on the interaction of rowing intensity and group. Rowing with higher intensity increased the cognitive costs in each age group [teens 1: *t*(9) = 5.18, *p* < 0.001; teens 2: *t*(9) = 4.89, *p* < 0.001; young: *t*(9) = 3.19, *p* = 0.005; middle-aged: *t*(9) = 2.72, *p* = 0.012].

For the DTCs in rowing speed, the mixed-design ANOVA with rowing intensity (2: easy vs. hard) as a within-subjects factor and group (4: teens 1, teens 2, young, and middle-aged) as a between-subjects factor revealed a significant main effect of rowing intensity, *F*(1, 36) = 7.93; *p* = 0.008; $\eta_{G}^{2}$ = 0.072. Motor costs were higher when rowing at a fast speed. There was no interaction of rowing intensity and age group, *F*(3, 36) = 1.80; *p* = 0.165; $\eta_{G}^{2}$ = 0.050. The main effect of age group reached significance, *F*(3, 36) = 4.80; *p* = 0.007; $\eta_{G}^{2}$ = 0.205. Bonferroni-corrected post-hoc comparisons of the four age groups for their overall rowing DTCs showed that the only comparison reaching significance was between teens 2 and young adults, *p* = 0.001, $M_{\mathrm{Diff}}$ = 5.971, 95%-CI[1.445, 10.497], with young adults showing the lowest level of motor costs.

### Discussion study 1

The findings of study 1 show that rowing on an ergometer while concurrently encoding word lists leads to considerable performance reductions: Participants encode fewer words while rowing, and they also show clear reductions in their rowing speeds, as well as their rowing regularity (see Supplementary material 2). Dual-task costs in both task domains (MoL and rowing) are even more pronounced when rowing with a faster speed. Although there are some interactions with the group factor, differences between groups in these effects tend to be small, and the overall pattern of dual-task performance reductions is consistent across groups.

Our findings contradict accounts that postulate improvements in episodic memory tasks due to an optimization of arousal levels via exercise bouts of low or intermediate intensities (Schmidt-Kassow et al., 2010, 2013, 2014; McMorris and Hale, 2012; Roig et al., 2013, 2016; Loprinzi et al., 2019). Instead, we found a linear decrease in memory performance from single-task to dual-task memory encoding, which is further increased when exercising with higher intensities. These findings are in line with dual-process theories (Shiffrin and Schneider, 1977; Furley et al., 2015). They also support a recent study by Longman et al. (2017), who reported cognitive and physical performance reductions in young rowers performing a rowing ergometer task combined with a free-recall task. In the Longman et al. study, participants had been instructed to produce maximum power output while rowing. This produced even higher costs in physical as compared to mental performance. Our findings indicate that rowing on an ergometer with the instruction of keeping up a pre-specified rowing speed is an attention-demanding task.

For study 2, the motor task consisted of a specific sequence of martial arts movements from Taekwondo (“practicing forms”), which is likely to be highly attention-demanding. Participants belonged to different skill groups. We predicted that the need to perform the MoL task concurrently with the Taekwondo movements will elicit cognitive and motor dual-task costs, which should be influenced by the expertise level of the participants.

## Study 2: Taekwondo

### Methods

#### Participants

Participants (*N* = 37) were recruited from five Taekwondo groups in the Saarland area. Of them, 28 participants practice classic Taekwondo, and the remaining nine participants practice Taekwondo in the World Taekwondo [WT] federation. All participants practiced Taekwondo as an amateur sport and had learned Taekwondo from different instructors. Three participants had experience with participation in Taekwondo form competitions. Considering graduation in Taekwondo, the lowest graduation in the sample is the 8th Kup (yellow belt) and the highest one is the 3rd Dan (black belt).

Participants were separated into three expertise groups based on their graduation in Taekwondo. The 10 participants with a yellow belt (8th and 7th Kup) formed the “relative beginners” group (7 men, 3 women), while the “intermediate level” group consisted of 17 athletes (8 men, 9 women) with a blue and a red belt (6th Kup–1st Kup). The third group (“experts”) consisted of 10 athletes (5 men, 5 women) with a black belt. Table 4 presents an overview of the background information of the three groups. All participants provided informed consent to participate in the study. The study was approved by the ethics committee of Saarland University.

**Table 4:** Background information about the three groups of Study 2 (Taekwondo).

Demographic variables		Expertise group		
		Beginners (yellow belt)	Intermediate (blue or red belt)	Experts (black belt)
*N* (men/women)		10 (7/3)	17 (8/9)	10 (5/5)
Age (years)	*M*	39	28	43
	*SD*	19	15	10
	*Range*	13–67	13–53	30–63
Experience in Taekwondo (years)	*M*	3.55	7.71	14.30
	*SD*	1.57	2.71	5.42
Weekly Taekwondo participation (minutes)	*M*	184	142	213
	*SD*	80	61	112
Digit symbol substitution test (correct items)	*M*	51.90	56	53.10
	*SD*	9.61	9.82	11.60

A statistical power analysis was performed for sample size estimation (GPower 3.1; Faul et al., 2007). Within-subjects designs and highly reliable measures increase statistical power (Brysbaert and Stevens, 2018; Rouder and Haaf, 2018; Zwaan et al., 2018; Brysbaert, 2019). We expected age- and expertise-related influences on dual-task decrements to be large (Leavitt, 1979; Schaefer and Scornaienchi, 2019; Amico and Schaefer, 2022; Schaefer and Amico, 2022). We conducted a power calculation using the G*3 Power software (Faul et al., 2007), with a significance level of 0.05. The power analysis focused on the interaction effect of expertise/age group and single- vs. dual-task performance decrements. We assumed the correlation among repeated measures to be high (*r* = 0.65; see also Schaefer and Scornaienchi, 2019). The analysis indicated that a medium-to-large effect size of *f* = 0.3 for four groups and two measurement occasions (single- vs. dual-tasking) would lead to an actual power of 0.96 with a total sample size of 40 participants.

#### Procedure

The study took place at the Dojang (training site to practice Taekwondo) of the Taekwondo group. Participants initially took part in an instruction session. After the assessment of demographic information and the Digit Symbol test, participants watched an instructional video tutorial on the method of Loci memory strategy, followed by two practice trials. For some participants, the instruction session was presented in an online format.

All MoL lists had been recorded in advance, and each list consisted of 15 location-word pairs that were presented with an ISI of 5 s via loudspeaker, resulting in trial lengths of 80 s. Encoding and recall were always cued.

For the testing session, list order was counterbalanced across participants, such that each list appeared equally often under single- and dual-task conditions. The experimenter checked the correctness of the recalled words (maximum score = 15 words). Participants performed the testing session either in casual sportswear or in their typical Taekwondo suit called *Dobok*.

At the beginning of the testing, participants were given 5 min to warm-up and were asked to perform the third form twice for practice. Table 5 presents the order of trials.

**Table 5:** Order of trials in study 2.

Cognitive single-task (MoL) Motor single-task (3rd form of Taekwondo) Dual-task trial 1 Dual-task trial 2 Motor single-task (3rd form of Taekwondo) Cognitive single-task (MoL)

In the single-task trials of MoL, participants performed the cognitive task while sitting without any concurrent activity. During the motor single-task trials, participants were filmed while performing the third form of Taekwondo. For the dual-task trials, participants performed the form while concurrently encoding the words. At the end of the recording, a signal was given by the experimenter, and participants had to stop their run immediately. They were asked to recall the presented words in the correct order. For dual-task trials, participants were instructed to focus on both the cognitive and the motor tasks as much as possible.

Following the testing sessions, the videos of the motor task were edited, anonymized, and then sent to the raters. There were no data exclusions, except for one trial of Taekwondo single-task for one participant, which had to be repeated due to technical difficulties. All data and analysis code can be made available upon request to the senior author. Data were analyzed using IBM SPSS Statistics 25 (IBM Corporation, Armonk, NY, United States). The study’s design and analyses were not pre-registered.

#### Apparatus and experimental task

##### Cognitive task–method of loci

In the cognitive task, participants were asked to encode and repeat words of a given list, similar to study 1. However, in the current study, there were only 15 words per list (instead of 20). The inter-stimulus interval was 5,000 ms.

##### Motor task–3rd form in Taekwondo

The motor task was the performance of the third Taekwondo form. A form of Taekwondo is a systematic, prearranged sequence of martial techniques used against one or more imaginary opponents (Minarik, 2014, p. 33). Participants perform a pre-specified sequence of stances, hand attacks, blocks, and kicks, which are to be presented with high precision. We chose the third form, named *To-San-Hyong* or *Taeguk sam jang,* because its difficulty level is optimal for the current study.

Participants were instructed to perform the form twice in a row. This specification was made for two reasons: First, the duration of the performance of the third form usually takes about 30 s. The recording of the MoL word lists takes about 80 s. To ensure that participants were moving until the recording was over, they were instructed to restart and repeat the form until the last pair of words had been presented. We expected that during the given 80 s, two complete runs of the form are possible. Participants were filmed while performing the form. All videos were edited so that the number of runs per video was equal under single- and dual-task conditions (2 runs each). There was only one exception for one slow participant who performed only one form under dual-task conditions.

The videos were rated independently by two raters, who were experienced scoring judges of official Taekwondo competitions. The raters are holders of the 3rd and the 4th Dan in Taekwondo and have been practicing Taekwondo for more than 30 years. Raters were not aware whether a specific form had been performed under single- or dual-task conditions. The rating was done using the *Jury’s Paper* of the DTU (2019), based on the following categories: *technique* and *presentation.* The domain *technique* refers to the accuracy of the martial techniques shown by the athlete, whereas *presentation* is measured by velocity and force, rhythm, and the expression of energy during the performance. The score on the *Jury’s paper* had been modified by doubling the possible points in each category. For two runs, the possible total score therefore was between 3 and 20 points, consisting of 0 to 8 points for *technique* and 3 to 12 points for *presentation*. For the one participant with only one valid run, the score was doubled.

#### Overview of analysis

The statistical analysis was performed via IBM SPSS Statistics 28 (IBM Corporation, Armonk, NY, United States).

We report ANOVAs on some background variables at the beginning of the Results section. The scores for the two trials in each domain (Taekwondo form: score in technique and score in presentation; score in MoL) were averaged across the respective conditions (single and dual).

The reliability of each of the three domains was tested by calculating Cronbach’s alpha. Mixed-design ANOVAs with expertise (3) as a between-subjects factor and condition (2: single- vs. dual-tasking) as a within-subjects factor were conducted for each of these dependent variables. In addition, dual-task costs were calculated. For MoL, a univariate ANOVA was conducted to reveal group differences in cognitive costs. For DTCs in Taekwondo, mixed-design ANOVAs with expertise (3) as a between-subjects factor and an evaluation category (2: technique, presentation) were conducted.

In addition, analyses for trial durations and the inter-rater reliabilities for the Taekwondo judges are reported in Supplementary materials 3, 4.

### Results

#### Participant background information

A one-way ANOVA with group (3) revealed a significant effect of group for the average age, *F*(2, 34) = 3.78, *p* = 0.033, $\eta_{p}^{2}$ = 0.182. The Bonferroni-corrected follow-up tests showed a significant difference only between intermediate-level athletes and experts (*p* = 0.045, *M_Diff* = 15.276, 95%-CI [0.26, 30.30]). There was no significant difference between the groups regarding their average weekly Taekwondo participation, *F*(2, 34) = 0.196, *p* = 0.823, $\eta_{p}^{2}$ = 0.011. Not surprisingly, there were significant differences between the groups regarding their mean experience in Taekwondo, *F*(2, 34) = 24.90, *p* < 0.001, $\eta_{p}^{2}$ = 0.594. The Bonferroni-corrected follow-up tests revealed significant differences between all three groups (beginners and experts: *p* < 0.001, *M_Diff* = 10.75, 95%-CI[6.87, 14.63]; intermediates and experts: *p* < 0.001, *M_Diff* = 6.594, 95%-CI[3.135, 10.053]; beginners and intermediates: *p* = 0.014, *M_Diff* = 4.156, 95%-CI[0.697, 7.615]). There were no differences between the groups in their cognitive speed, *F*(2, 34) = 0.568, *p* = 0.572, $\eta_{p}^{2}$ = 0.032, as measured with the Digit-Symbol Substitution test (Wechsler, 1981).

#### Cognitive task–MoL

**Figure 5:**
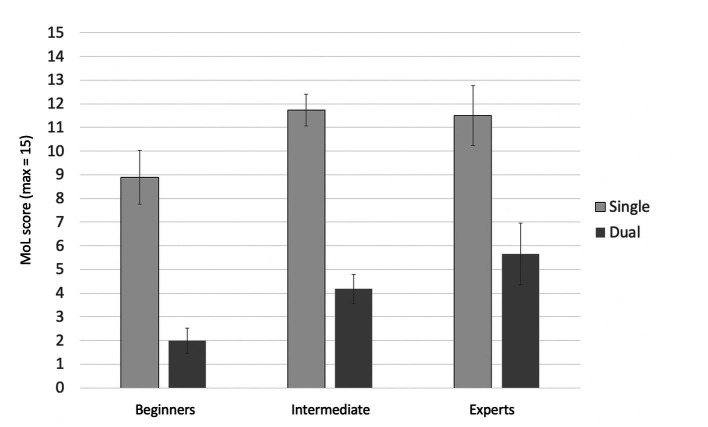
Memory performances by single- and dual-task condition and group, Study 2 (Taekwondo). For the single task, memory encoding took place while sitting. For the dual task, participants performed the third form of Taekwondo during encoding. Error bars = SE mean.

Cronbach’s alpha for the four trials of MoL was high, *α* = 0.86. The mixed-design ANOVA with expertise (3) as a between-subjects factor and single- vs. dual-tasking (2) as a within-subjects factor showed a significant main effect of single- versus dual-tasking, *F*(1, 34) = 216.41; *p* < 0.001; $\eta_{G}^{2}$ = 0.545. Participants recalled fewer words under dual-task conditions. The main effect of expertise also reached significance, *F*(2, 34) = 3.59, *p* = 0.039, $\eta_{G}^{2}$ = 0.146. However, there was no significant interaction between expertise and single- vs. dual-tasking, *F*(2, 34) = 1.25, *p* = 0.300, $\eta_{G}^{2}$ = 0.013. A Bonferroni-corrected *post hoc* analysis revealed no significant differences in the means of the score in MoL between the three expert groups (beginners and experts: *p* = 0.055; experts and intermediate: *p* = 1.000; 2 and 1: *p* = 0.097), but the difference between beginners and experts showed a trend in favor of the experts. The pattern of findings is depicted in Figure 5.

#### Motor task–3rd form in Taekwondo

The results concerning the Taekwondo form are the average of the ratings of both raters. Cronbach’s alpha for the four trials was high for both domains: *α* = 0.91 (technique) and *α* = 0.97 (presentation).

#### Technique

**Figure 6:**
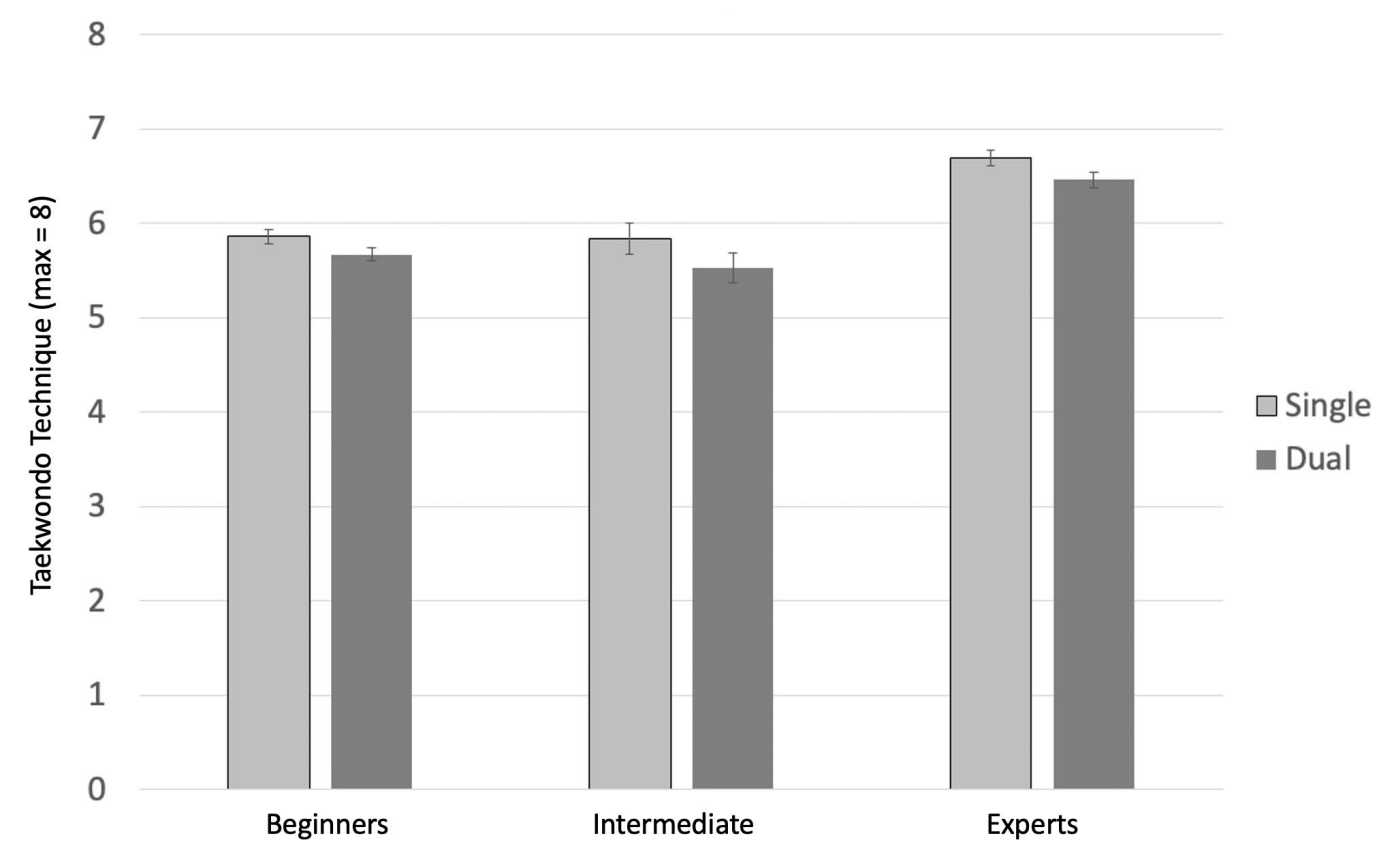
Mean scores in Taekwondo technique by single- and dual-task condition and group. Error bars = SE mean.

The findings in the category *technique* are shown in Figure 6. The ANOVA with expertise (3) as a between-subjects factor and single- vs. dual-tasking (2) as a within-subjects factor showed a significant main effect of single- vs. dual-tasking, *F*(1, 34) = 46.86; *p* < 0.001; $\eta_{G}^{2}$ = 0.058. The technique was better in the single-task condition. The main effect of expertise also reached significance, *F*(2, 34) = 12.39, *p* < 0.001, $\eta_{G}^{2}$ = 0.412. However, there was no significant interaction between expertise and single- vs. dual-tasking, *F*(2, 34) = 1.51, *p* = 0.236, $\eta_{G}^{2}$ = 0.038. A Bonferroni-corrected post-hoc analysis revealed a significant difference in the means of the score in *technique* between beginners and experts (*p* = 0.001, $M_{\mathrm{Diff}}$ = 0.80, 95%-CI[0.28, 1.33]), as well as between intermediates and experts (*p* < 0.001, $M_{\mathrm{Diff}}$ = 0.89, 95%-CI[0.42, 1.35]), but no significant difference between beginners and intermediates (*p* = 1.000). As expected, experts (holders of a black belt) gained significantly higher scores in *technique* than the other two groups.

#### Presentation

**Figure 7:**
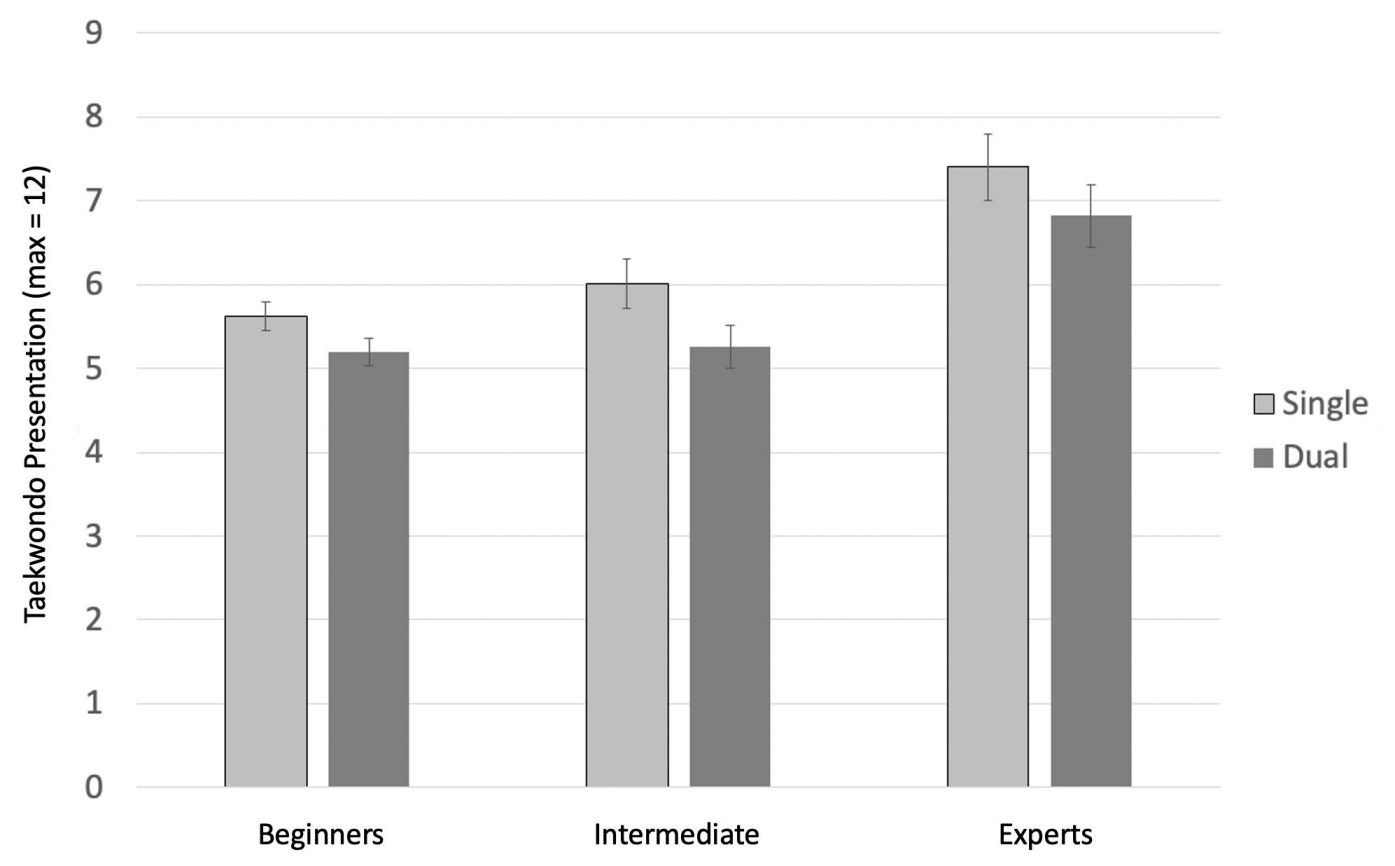
Mean scores in Taekwondo presentation by single- and dual-task condition and group. The figure depicts the average score in presentation achieved by each expert group depending on the respective condition (single and dual). Error bars = SE mean.

Figure 7 presents the findings for presentation. For presentation, the ANOVA with expertise (3) as a between-subjects factor and condition (2) as a within-subjects factor showed a significant main effect of single- vs. dual-tasking, *F*(1, 34) = 63.91; *p* < 0.001; $\eta_{G}^{2}$ = 0.076. Presentation suffered under dual-task conditions. The main effect of expertise also reached significance, *F*(2, 34) = 8.84, *p* < 0.001, $\eta_{G}^{2}$ = 0.333. Yet, there was no significant interaction of expertise and condition, *F*(2, 34) = 1.91, *p* = 0.164, $\eta_{G}^{2}$ = 0.005. A Bonferroni-corrected post-hoc analysis revealed a significant difference in the means of the score in *presentation* between beginners and experts (*p* = 0.002, $M_{\mathrm{Diff}}$ = 1.70, 95%-CI[0.56, 2.83]), as well as between intermediates and experts (*p* = 0.003, $M_{\mathrm{Diff}}$ = 1.50, 95%-CI[0.46, 2.48]), but no significant difference between beginners and intermediates (*p* = 1.000). Corresponding to expectations, experts (black belts) achieved significantly higher scores in presentation than the other groups.

#### Dual-task costs

Dual-task costs for each performance domain were calculated based on the formula presented in study 1. The right-hand side of Figure 4 presents the DTCs for MoL (Figure 4B) and for the two Taekwondo dimensions, technique and presentation (Figure 4D).

Expertise groups did not differ significantly in their cognitive DTCs, *F*(2, 34) = 2.39, *p* = 0.107, $\eta_{p}^{2}$ = 0.123.

To examine the difference in the average costs in the Taekwondo domains, a mixed-design ANOVA was calculated, with expertise (3) as a between-subjects factor and an evaluation category (2: technique, presentation) as a within-subjects factor. The analysis revealed a significant main effect of category, *F*(1, 34) = 60.72, *p* < 0.001, $\eta_{G}^{2}$ = 0.212. The costs of *presentation* were significantly higher than those of *technique.* The main effect of expertise did not reach significance, *F*(2, 34) = 2.7, *p* = 0.082, $\eta_{G}^{2}$ = 0.119. In addition, there was no significant interaction of expertise and category, *F*(2, 34) = 2.21, *p* = 0.125, $\eta_{G}^{2}$ = 0.019.

Supplementary material 5 reports scatterplots for the cognitive and motor DTCs for both studies.

### Discussion study 2

The findings of study 2 show that doing a form in Taekwondo requires a lot of attention: Participants in each skill level show considerable performance reductions in the concurrent memory task, with reductions between almost 60% in the most advanced athletes (black belt) and about 80% in the relative beginners (yellow belt). The quality of the movement is also reduced, as shown by the costs in technique and presentation. The fact that expertise groups did not differ systematically in the extent of the performance decrements may be related to power issues since the study only tested 37 athletes. Consistent with expectations, holders of black belts showed significantly better absolute performances in Taekwondo than the other two groups. Nevertheless, based on the current dataset, even being the holder of a black belt did not protect the athletes from failing to maintain their cognitive and motor performances under dual-task conditions (see also Supplementary material 5).

Study 2 also revealed that motor dual-task costs were higher in the performance category *presentation* than in *technique*, irrespective of graduation. This indicates that encoding words using a mental imagery technique still enables participants to perform the movements in the correct order (*technique*), but movement velocity and force, rhythm, and expression of energy during the performance (*presentation*) suffer more strongly.

## General discussion

We investigated whether performing a skilled motor task like rowing on an ergometer (study 1) or presenting a pre-specified sequence of martial arts movements (study 2) results in performance reductions in a concurrent episodic memory task (MoL) and whether dual-task costs are moderated by motor expertise. Subjects in both studies had at least some experience with the respective motor skills, and some were relatively advanced (i.e., the young adult rowers in study 1 and the holders of the black belts in study 2). Nevertheless, performance in both the cognitive and the motor tasks decreased significantly in all groups, indicating that the attentional demands of well-practiced motor skills are considerable.

Previous studies had used MoL memory encoding in combination with different motor tasks (while walking on easy and complex narrow tracks with and without obstacles: Li et al., 2000, 2001; while balancing on an ankle-disc board: Schaefer et al., 2008; while rotating a fidget spinner, doodling, or tracing: Amico and Schaefer, 2020; while doing fast or slow squats: Amico et al., 2023). Participants were children, young, or older adults. Performing a motor task concurrently reduced MoL performances in most studies, with the only exceptions being while kneading a stress ball (Amico and Schaefer, 2020) or while walking on a simple track without obstacles (Li et al., 2000, 2001), both in young adults only.

The highest MoL costs in previous studies occurred when encoding took place while tracing symbols (about 40% in young adults; Amico and Schaefer, 2020), while walking on complex tracks with obstacles (about 40% in older adults; Li et al., 2000, 2001), or while doing squats with every second word (about 35% in young adults; Amico et al., 2023). The cognitive costs of the current studies are considerably higher than that, with about 60% during fast rowing in study 1 and almost 80% for the relatively less-experienced holders of yellow belts in study 2 (see Figure 4). This indicates that the motor tasks of fast ergometer rowing and doing forms in Taekwondo require substantial cognitive processing resources, such as attention and working memory capacity (Shiffrin and Schneider, 1977; Kahneman, 2011; Evans and Stanovich, 2013; Furley et al., 2015).

It may seem somewhat surprising that ergometer rowing reduced cognitive performances considerably since bouts of low- or intermediate-intensity exercise even exerted beneficial effects on memory performance in previous studies (e.g., Schmidt-Kassow et al., 2010, 2013, 2014; see also Loprinzi et al., 2019, for a review). However, concurrent motor tasks were self-paced in these contexts and were not framed as a secondary task. For the current study, participants had been instructed to keep up their predetermined rowing speeds, which requires closely and continuously monitoring the ergometers’ display. It would be interesting to disentangle the cognitive requirements of monitoring the ergometer display from the effects of rowing as a motor activity. Therefore, future studies should include a condition in which participants do not receive any online feedback about their rowing speed during the trial but are still instructed to keep up their rowing speed under dual-task conditions. In addition, memory recall in our study took place immediately after the end of the encoding phase. The studies finding positive effects of exercise on memory encoding often assessed memory recall 1 or 2 days later (Schmidt-Kassow et al., 2013, 2014). In the dual-task trials of our study, we cannot rule out that physical fatigue from intense rowing may have led to additional decrements in recall performance. Future research should, therefore, systematically vary the time delay between encoding and recall.

Both studies also found decrements in motor performances caused by the cognitive task: Rowing became slower and more irregular in study 1, and the quality of the Taekwondo presentation suffered. Motor costs were not as high as in cognition (see Figure 4), which may be due to motor tasks being prioritized by the athletes (Gibson, 1979; Schaefer, 2014; Plummer and Eskes, 2015; Amico and Schaefer, 2022). Differential-emphasis instruction can shed further light on the interindividual differences in the ability to shift one’s focus of attention strategically (Kramer et al., 1995; Li et al., 2005). In addition, providing participants with more practice in the dual-task situation may reduce their cognitive–motor dual-task costs (Schumacher et al., 2001). However, since every participant of the current study showed costs in at least one of the two tasks (for details, see Supplementary material 5), it is an open question whether extended practice would eliminate their dual-task costs entirely.

From an applied perspective, it is interesting that a skill like rowing seems not to be fully automatized, even in elite rowers. Rowing times in the hard conditions were reduced considerably under dual-task conditions (see Figure 2). In addition, the increase in rowing irregularity is very strong in the current study (see Figure 3), even in the group with the highest performance level. Rowers often perform in groups, which implies that they need to keep their balance on the boat, while at the same time coordinating their rowing with several other athletes. Each of these aspects may require cognitive resources. Therefore, the cognitive demands of the sport are likely to be higher than expected. The fact that only a few of the athletes use entertainment, such as listening to music while training, also hints in this direction (see Table 1). Future research should address each of these aspects separately with systematic experimental manipulations. In addition, neuropsychological measures can add to our understanding of dual-task interference (Leone et al., 2017; Mac-Auliffe et al., 2021).

### Limitations and future directions

Both studies would have profited from larger sample sizes. For the cognitive DTCs of study 2, there is a trend in favor of smaller costs for expert athletes. Larger samples should allow for a clearer picture on expertise differences in cognitive–motor dual-tasking. In addition, study 1 should have included a 180-s single-task rowing trial for the hard speed as well to further support the claim that changes in rowing speed under dual-task conditions are not due to physical fatigue.

Motor expertise is not the only factor that can influence cognitive–motor performance trade-offs. There is a rich literature documenting aging-related changes to perform cognitive and motor tasks concurrently, mainly in the context of everyday locomotor activities such as walking or balancing (for reviews, see Beurskens and Bock, 2012; Li et al., 2018; Wollesen and Voelcker-Rehage, 2019; Kahya et al., 2020). To the best of our knowledge, there are few studies that have recruited skilled athletes of advanced age. Study 1 included rowers in middle adulthood, who showed similar performance decrements as the other groups. Findings in even older adults would be very informative. Future research should investigate whether motor expertise may even counteract aging-related decrements (see also Krampe et al., 2014), allowing older individuals to keep up their cognitive and motor performance in challenging dual-task situations.

Future studies should also disentangle different aspects of the motor skill (e.g., motor planning vs. motor execution) and their mutual interference with cognitive tasks (Logan and Fischman, 2011, 2015; Spiegel et al., 2013), which requires a more fine-grained analysis of the temporal dynamics during dual-tasking. As Koch et al. (2018) pointed out, continuous tasks and aggregated performance measures demonstrate dual-task interference on a “macrolevel,” making it difficult to reveal task-scheduling and switching processes on a “microlevel.” When performing a Taekwondo form, athletes probably rely on the chunking of movements that are called upon and then executed in response to specific attacks (either imagined or real). If the presentation of words for the MoL task coincides with the preparation of specific movement chunks, mutual interference may be higher than during the execution of the movement. Future research should address these temporal dynamics.

## Conclusion

The current set of studies combined an episodic memory task with the performance of a continuous, cyclic motor skill requiring primarily strength and endurance (rowing, study 1) or an elaborate motor skill with high demands on timing, movement accuracy, and coordination (Taekwondo forms, study 2). Both studies compared athletes from different ability groups in the extent to which both cognitive and motor performances suffered from the concurrent task. Although higher-level athletes outperformed others in motor skills under single-task conditions, proportional dual-task costs were similar across skill levels. Costs occurred in each individual and in the motor as well as the cognitive domain. This indicates that even well-practiced motor tasks require cognitive resources.

## Data availability statement

The raw data supporting the conclusions of this article will be made available by the authors, without undue reservation.

## Ethics statement

The studies involving humans were approved by Ethics Committee of Saarland University. The studies were conducted in accordance with the local legislation and institutional requirements. Written informed consent for participation in this study was provided by the participants’ legal guardians/next of kin.

## Author contributions

KM and SS contributed to the conception and design of study 1 (rowing) and performed the statistical analysis for study 1. AM, MK, and SS contributed to the conception and design of study 2 (Taekwondo) and performed the statistical analysis for study 2. MK was involved in the analyses for Supplementary material 4. SS wrote the first draft of the manuscript. AM wrote sections of the manuscript (study 2). All authors contributed to the article and approved the submitted version.
