# Supplementary material for: Mutual interference between memory encoding and motor skills: the influence of motor expertise
Source: Front Psychol. 2023 Dec 15;14:1196978. doi: 10.3389/fpsyg.2023.1196978 (PMC10755016; doi:10.3389/fpsyg.2023.1196978)
Supplement: Supplementary file 1 [file Data_Sheet_1.pdf]

Supplementary Material 1: Heart Rates While Rowing

Mutual Interference between Memory Encoding and Motor Skills: The Influence of Motor Expertise

Annalena Monz, Kathrin Morbe, Markus Klein & Sabine Schaefer*

*** Correspondence:** sabine.schaefer@uni-saarland.de

Table S 1

Heart Rates While Rowing in the Four Groups of Study 1

Age Group		Teenager 1	Teenager 2	Young Adults	Middle-Aged Adults
Heart Rate Rowing Only, Easy	*M*	131.8	139.7	129.9	119.9
	SD	8.2	6.9	6.7	7.9
Heart Rate Rowing Plus MoL, Easy	*M*	141.0	144.8	139.5	123.7
	SD	8.6	6.6	8.9	7.3
Heart Rate Rowing Plus MoL, Hard	*M*	166.9	172.5	173.0	144.7
	SD	9.1	5.3	5.2	7.4

Note. Heart rates are measured in beats per minute. “Heart Rates Rowing Only, Easy” is the average of the heart rates assessed in sessions 1 and 4. “Heart Rate Rowing Plus MoL, Easy” and “Heart Rate Rowing Plus MoL, Hard” refer to the average heart rates of the entire trial (single plus dual-task segment).
