# Supplementary material for: Mutual interference between memory encoding and motor skills: the influence of motor expertise
Source: Front Psychol. 2023 Dec 15;14:1196978. doi: 10.3389/fpsyg.2023.1196978 (PMC10755016; doi:10.3389/fpsyg.2023.1196978)
Supplement: Supplementary file 2 [file Data_Sheet_2.pdf]

Supplementary Material 2: Fluctuations in Rowing Times Over Trial Segments

Mutual Interference between Memory Encoding and Motor Skills: The Influence of Motor Expertise

Annalena Monz, Kathrin Morbe, Markus Klein & Sabine Schaefer*

*** Correspondence:** sabine.schaefer@uni-saarland.de

Dual-tasking led to an increase in average rowing times. The software of the rowing ergometers recorded rowing performances for each 10-second-segment of the trial, which allows to plot changes in rowing performance over time. Figures S 1 to S 4 present these patterns for each age group.

Figure S 1

Rowing Times by Segment for the Younger Teenagers, Study 1

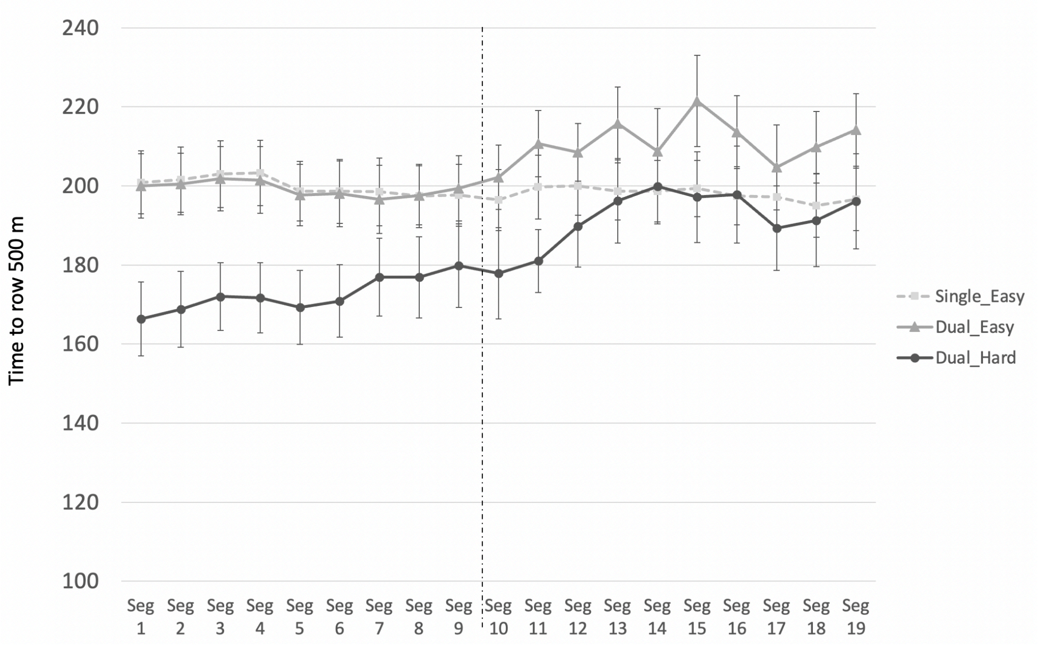

Figure S 2

Rowing Times by Segment for the Older Teenagers, Study 1

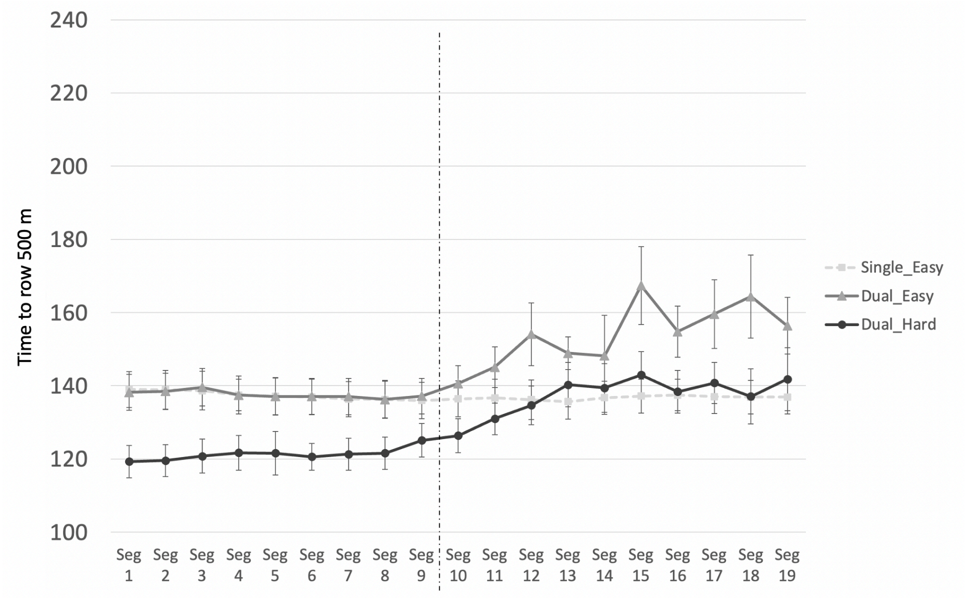

Figure S 3

Rowing Times by Segment for the Young Adults, Study 1

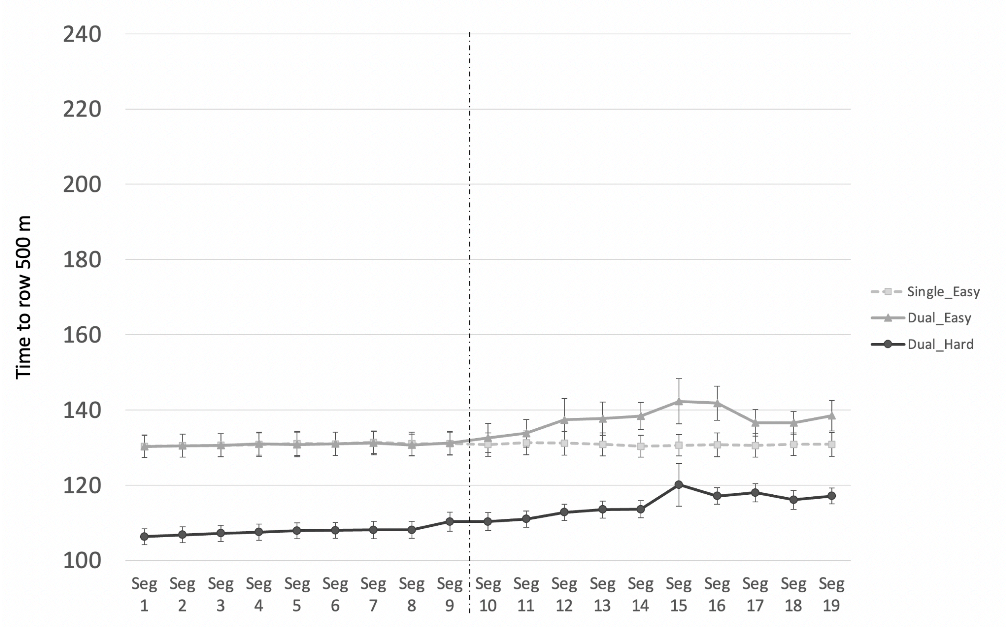

Figure S 4

Rowing Times by Segment for the Middle-Aged Adults, Study 1

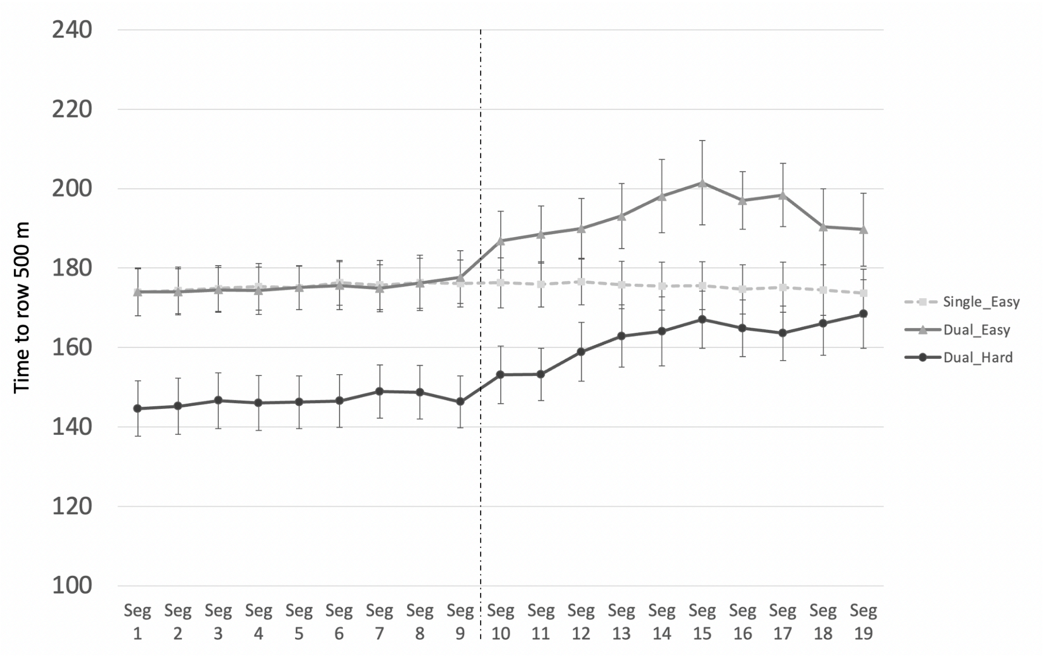

Note. Each trial consists of 19 segments. The first half of each "dual-task" trial is assessed without any concurrent cognitive task. Stimulus presentation for the MoL task started in segment 10. "Single-Easy" refers to the trials assessed in sessions 1 and 4, which were performed without any cognitive task. Error bars = SE mean.

The Figures S 1 to S 4 show that rowing became slower and less regular under dual-task conditions, and this happened in all age groups and in both rowing speeds. Note that sessions 1 and 4 also assessed a 180-second easy speed rowing trial without any cognitive task ("single-easy"). There were no performance changes in these trials in any of the groups, indicating that changes in rowing speed after segment 10 are due to the cognitive challenge of the MoL task, and not due to fatigue effects.
