# Supplementary material for: Mutual interference between memory encoding and motor skills: the influence of motor expertise
Source: Front Psychol. 2023 Dec 15;14:1196978. doi: 10.3389/fpsyg.2023.1196978 (PMC10755016; doi:10.3389/fpsyg.2023.1196978)
Supplement: Supplementary file 3 [file Data_Sheet_3.pdf]

Supplementary Material 3: Durations of Individual Taekwondo Runs Under Single-and Dual-Task Conditions

Mutual Interference between Memory Encoding and Motor Skills: The Influence of Motor Expertise

Annalena Monz, Kathrin Morbe, Markus Klein & Sabine Schaefer*

*** Correspondence:** sabine.schaefer@uni-saarland.de

In addition to the rating of the forms, we also examined the duration of a run. It was assumed that in dual-task trials, athletes with a lower graduation in Taekwondo would take a significantly longer time to perform the form than the experts (black belts), which may be one of the reasons for achieving a lower score in presentation.

Figure S1

Mean Duration of Form Presentation

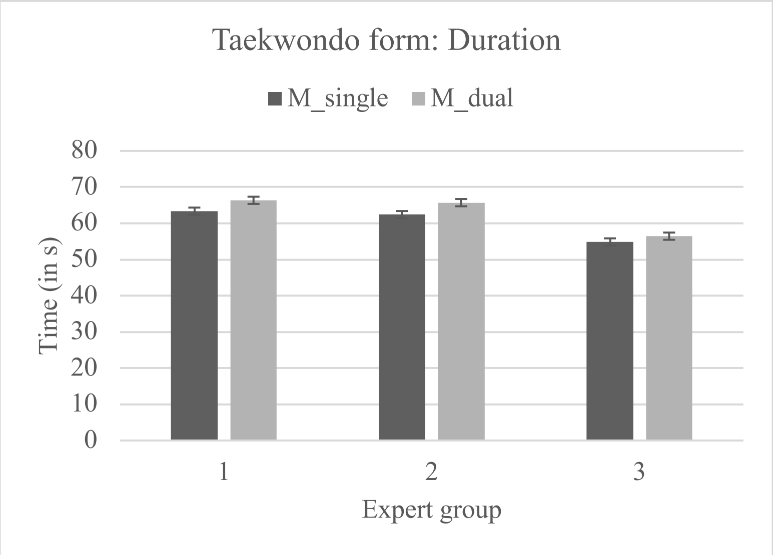

Note. Mean times for two runs of the form for each expert group (1: beginners, 2: intermediate, 3: experts) depending on the respective condition (single, dual). Error bars = SE mean

To examine this, a mixed-design ANOVA with expertise (3) as between-subjects factor and condition (2) as within-subjects factor was conducted. The analysis was based on the total time for two runs. The mixed-design ANOVA revealed a significant main effect of single- versus dual-tasking, $F(1, 34) = 6.05$; $p = .019$; $\eta_p^2 = .151$. Participants took longer to finish a form under dual-task conditions. The main effect of expertise did not reach significance, $F(2, 34) = 2.63$, $p = .087$, $\eta_p^2 = .134$. Also, there was no significant interaction of expertise and condition, $F(2, 34) = .24$, $p = .786$, $\eta_p^2 = .014$. The results are illustrated in Figure S 1.
