# Supplementary material for: Mutual interference between memory encoding and motor skills: the influence of motor expertise
Source: Front Psychol. 2023 Dec 15;14:1196978. doi: 10.3389/fpsyg.2023.1196978 (PMC10755016; doi:10.3389/fpsyg.2023.1196978)
Supplement: Supplementary file 4 [file Data_Sheet_4.pdf]

Supplementary Material 4: Interrater Reliability of Taekwondo Raters

Mutual Interference between Memory Encoding and Motor Skills: The Influence Motor of Expertise

Annalena Monz, Kathrin Morbe, Markus Klein & Sabine Schaefer*

*** Correspondence:** sabine.schaefer@uni-saarland.de

Table S 1

ICCs of the Evaluation Categories of the Taekwondo Form

		95%-Confidence Interval	
Evaluation Category	ICC	Lower Limit	Upper Limit
Total score_single	0.838	0.668	0.919
Total score_dual	0.845	0.685	0.923
Technique_single	0.622	-0.220	0.866
Technique_dual	0.576	-0.228	0.843
Presentation_single	0.849	0.704	0.923
Presentation_dual	0.852	0.712	0.924

*Significance: $p < .001$

To assess conformity among both raters, interrater reliability was evaluated by calculating the intraclass correlation coefficient [ICC] (see Koo & Li, 2016). One participant was excluded from the dataset, because each of the raters declared one of the runs of this participant as invalid. The results concerning the ICCs of each evaluation category of the form across the two conditions (single, dual) are illustrated in Table S 1. Note that the Table also includes total scores, consisting of both rating categories (Technique and Presentation).

The evaluation of the ICCs was based on the interpretation of the ICC by Cicchetti (1994) and Koo and Li (2016) as shown in Table S 2. According to Cicchetti (1994), the ICCs of total score and presentation are excellent, while they are good based on the interpretation of Koo and Li (2016, p. 158). The ICC of technique under the single-task condition is good, and the one under the dual-task condition is moderate (see Cicchetti, 1994, p. 286). According to Koo and Li (2016, p. 158) the ICCs of technique are moderate for both conditions. In conclusion, the ICC values of the raters of the current study are high with regard to a comparable data set of an official federal ranking tournament (see Klein et al., 2014).

Table S 2

Interpretation of the ICC

Reliability	Cicchetti (1994)	Koo and Li (2016)
poor	< 0,4	< 0,5
moderate	0,4–,6	0,5–0,75
good	0,6–0,75	0,75–0,9
excellent	> 0,75	> 0,9

When interpreting the Taekwondo performance ratings in the current study, it should be kept in mind that common judgement biases such as sequence effects or self-fulfilling prophecies could have influenced the ratings. Participants were free to either wear normal sportswear or their typical dobok and belt. For participants in a dobok, raters could see the graduation of the participant (as indicated by the color of the belt). Consequently, the raters may expect black belts to perform better than athletes with a lower graduation in Taekwondo. Therefore, black belts could either be evaluated more strictly, or - on the contrary- be credited with more points because they are considered to have a high expertise, independent from their actual performance. Future research with the current paradigm should avoid this, by asking all participants to wear normal sportswear, without any visible graduation.

References

Cicchetti, D. V. (1994). Guidelines, criteria, and rules of thumb for evaluating normed and standardized assessment instruments in psychology. *Psychological Assessment, 6*(4), 284–290. https://doi.org/10.1037/1040-3590.6.4.284

Klein, M., Frenger, M., & Sommer, T. (2014). Interrater-Reliabilität der Punktrichterentscheidungen im Taekwondo-Formenlauf (Interrater-Reliability of Performance Judgements in Martial Arts). In S. Liebl & P. Kuhn (Hrsg.), *Menschen im Zweikampf – Kampfkunst und Kampfsport in Forschung und Lehre 2013: 3. Internationales Symposium „Kampfkunst und Kampfsport" vom 7.-9. November 2013 in Erlangen* (p. 209–215). Hamburg: Feldhaus.

Koo, T. K., & Li, M. Y. (2016). A guideline of selecting and reporting intraclass correlation coefficients for reliability research. *Journal of Chiropractic Medicine, 15*(2), 155–163. https://doi.org/10.1016/j.jcm.2016.02.012
