# Supplementary material for: Mutual interference between memory encoding and motor skills: the influence of motor expertise
Source: Front Psychol. 2023 Dec 15;14:1196978. doi: 10.3389/fpsyg.2023.1196978 (PMC10755016; doi:10.3389/fpsyg.2023.1196978)
Supplement: Supplementary file 5 [file Data_Sheet_5.pdf]

Supplementary Material 5: Scatterplots for Dual-task Costs

Mutual Interference between Memory Encoding and Motor Skills: The Influence of Motor Expertise

Annalena Monz, Kathrin Morbe, Markus Klein & Sabine Schaefer*

*** Correspondence:** sabine.schaefer@uni-saarland.de

Performance trade-offs between the motor and the cognitive task can be revealed by comparing the dual-task costs for the two domains. Figures S1 and S2 shows the scatterplots for the two studies.

Figure S 1

Scatterplot for Dual-Task Costs by Domain, Study 1

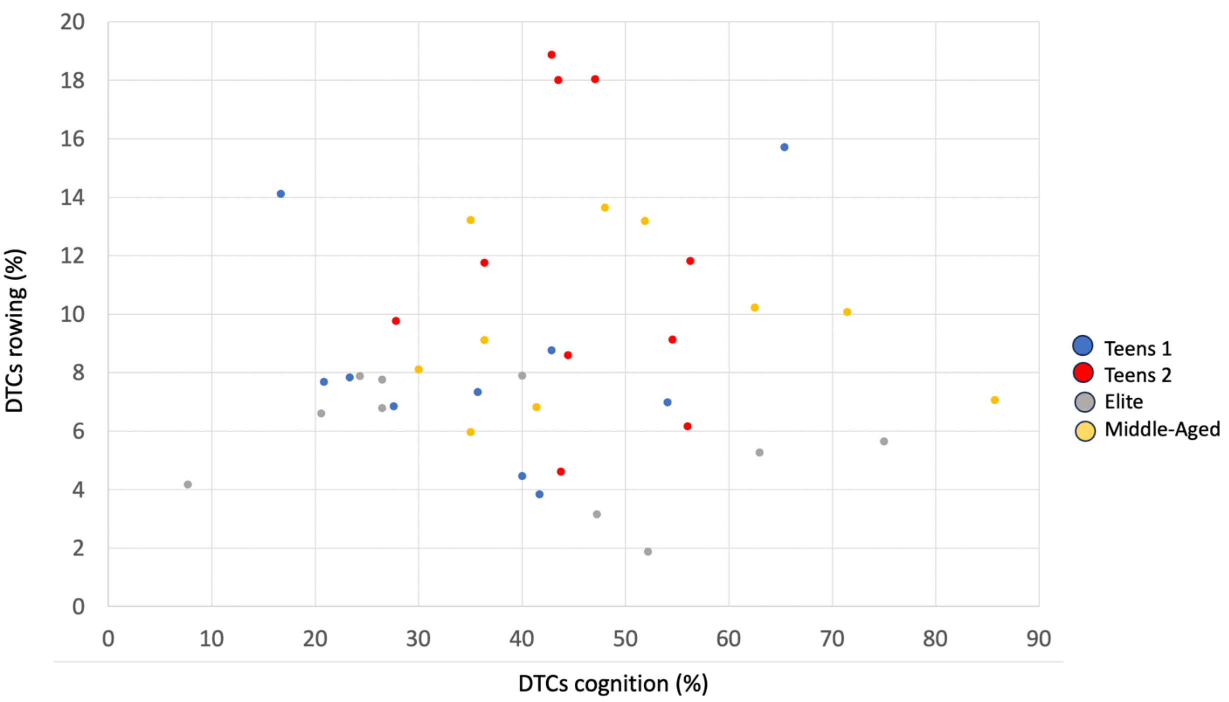

Note. Each participant's proportional dual-task costs have been averaged across rowing intensities (easy and hard). Colors denote the group.

Figure S 2

Scatterplot for Dual-Task Costs by Domain, Study 2

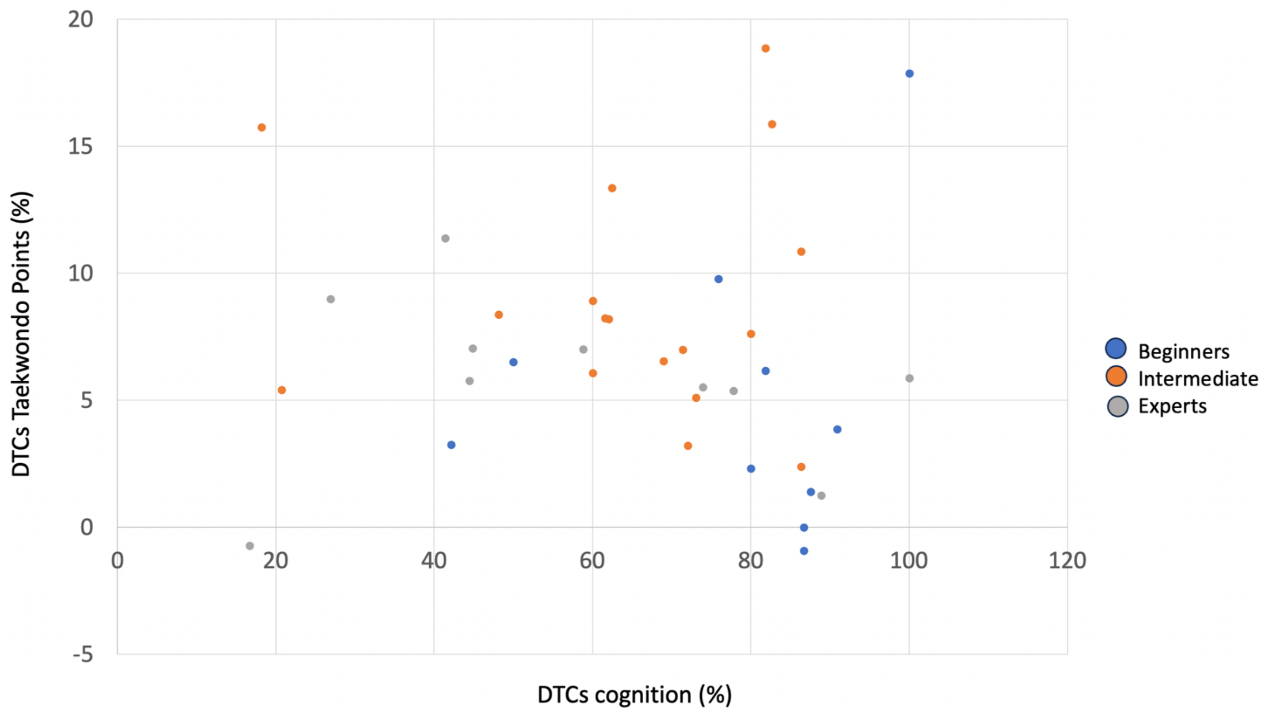

Note. Proportional dual-task costs in Taekwondo refer to the points. Colors denote the group.

For both studies, there is no significant correlation between cognitive and motor costs (study 1: $r(40) = .069$, $p = .671$; study 2: $r(37) = -.049$, $p = .775$). A visual inspection of the color-coded groups reveals no clear between-group differences in focusing more on one task over the other. In addition, there are no individuals that can perform both concurrent tasks without any costs in the current studies. This is different from a previous study by Watson & Strayer (2010), who found that a small

group of their participants (“supertaskers”) was able to drive in a stimulator while performing a demanding auditory version of the operation span (OSPAN) task without any costs.

References

WATSON, J. M. & STRAYER, D. L. 2010. Supertaskers: Profiles in extraordinary multitasking ability. *Psychonomic Bulletin & Review,* 17, 479-485.
